# Intensification and optimization of biodiesel production using microwave-assisted acid-organo catalyzed transesterification process

**DOI:** 10.1038/s41598-020-77798-1

**Published:** 2020-12-04

**Authors:** Moina Athar, Sadaf Zaidi, Saeikh Zaffar Hassan

**Affiliations:** 1 Department of Petroleum Studies, Zakir Husain College of Engineering and Technology, Faculty of Engineering and Technology, Aligarh Muslim University, Aligarh, India; 2 Department of Post Harvest Engineering and Technology, Faculty of Agricultural Sciences, Aligarh Muslim University, Aligarh, India

**Keywords:** Biodiesel, Chemical engineering

## Abstract

To produce biodiesel cost-effective, low-cost, high free fatty acid (FFA) oil feedstock is desirable. But high FFA creates difficulty during the base-catalyzed transesterification process by yield loss due to the formation of soap. However, these problems are overcome by the use of an acid catalyst. The acid catalysts can directly convert both triglycerides and FFAs into biodiesel without the formation of soaps or emulsions. The shortcomings of mostly used inorganic acids are that they work well for esterification of FFA present in low-cost oil, but their kinetics for transesterification of triglycerides present in oils is considerably slower. Corrosion of equipment is another major problem associated with an inorganic acid catalyst. The usage of an organic acid catalyst of the alkyl benzene sulfonic type, like 4-dodecyl benzene sulfonic acid (DBSA) minimizes these disadvantages of inorganic acid-catalyzed transesterification. The aim of the present investigation was to reduce the reaction time of transesterification of triglycerides further by using microwaves as a heating source in the presence of DBSA catalyst to achieve higher conversions under mild operating conditions. To optimize the transesterification variables for the higher conversion of biodiesel, the response surface methodology was employed to design the experiment. By using the DBSA catalyst under microwave heating at a temperature of 76 °C, conversion close to 100% in only 30 min of reaction time was obtained using a 0.09 molar ratio of catalyst to oil and 9.0 molar ratio of methanol to oil. A modified polynomial model was developed and was adequately fitted with the experimental data and could be used for understanding the effect of various process parameters. The catalyst to oil molar ratio and reaction temperature created a stronger effect on the biodiesel production than that exhibited by the methanol to oil molar ratio. It was observed that the microwave heating process outperformed conventional heating, providing a rapid, easy method for biodiesel synthesis from triglycerides in the presence of DBSA, an organic acid catalyst. The produced biodiesel was of good quality, as all the properties were within the prescribed limits of the ASTM D6751 standard.

## Introduction

As the present reserves of fossil fuels are gradually decreasing, it is obvious that their prices will continue to grow, and their imports will put an increasing burden on national economies, which will ultimately provide an opportunity for the growth of renewable energy usage. Amongst renewable energy resources, biodiesel is getting progressively more importance due to its relatively simple methods of production. Biodiesel consists of alkyl esters of fatty acids produced by the transesterification of triglycerides (TG) or esterification of free fatty acids (FFAs) using alcohol with or without a catalyst. Methanol is the most common alcohol used for making biodiesel. Fatty acid esters that are produced by methanol are known as fatty acid methyl esters (FAME). Normally, a homogeneous, alkali-catalyzed transesterification process is used industrially because of faster kinetics and economical feasibility^1^. However, the major problem of this technique is its susceptibility towards impurities, particularly water and FFA content^2–5^. To decrease the cost of production, less-costly, high FFA oil should be utilized rather than refined oil, for which base catalyst is not suitable. As the low-cost non-edible oils contain a high amount of FFA together with triglycerides, a two-step esterification and transesterification process is usually essential, i.e., the free fatty acids are first changed to the alkyl esters using acid-catalyst by esterification, whilst the conversion of triglycerides by alkaline catalyst into alkyl esters is the second step^3,4,6,7^. Unfortunately, in the first step, FFA may often not decrease effectively because of the large amount of water produced in the esterification reaction as a by-product. In such a situation, an additional amount of alcohol and sulphuric acid is usually added to the feedstock three times (three-step pre-esterification)^8^. The water produced during the pre-treatment phase needs removal, which entails high capital investment, thereby limiting the usage of the process. Further, the time required for esterification is also considerable due to the sluggish reaction rate which increases as the amount of FFA increases. Even this will need additional energy to recycle the extra alcohol used^9^. Another approach is the acid-catalyzed one-step method which promotes both the esterification and the transesterification reactions at the same time^10,11^. A few mineral acid catalysts (inorganic) such as phosphoric acid, sulfuric acid, or hydrochloric acid are less susceptible to FFA and can all together carry out both esterification and transesterification reactions without any problem of phase separation or soap formation^12^. Although these catalysts support a high yield of esters and fast reaction kinetics for esterification of FFA present in low-cost non-edible oils but require higher pressures, high reaction temperatures, and much slower reaction kinetics (about 4000 times) for transesterification of triglycerides present in oils^4,13–15^. The slower reaction rate of transesterification by these catalysts may be due to the reaction in the alcohol phase where the concentration of oil is low because of the slow diffusion of viscous oil in the alcohol phase. So the rate of reaction is regulated by the rate of mass transfer between the oil and alcohol phases^16^. Apart from the slow rates of reaction, transesterification with acid catalyst also needs corrosion-resistant vessels to tolerate the corrosion of mineral acids, which enhances the capital and operating costs of the production process^12^. The challenges mentioned above can be resolved by the use of less corrosive but strong, organo-sulfonic acids. One of these types of organic acid catalysts used by some researchers is 4-dodecyl benzene sulfonic acid (DBSA)^2,16,17^. DBSA has a sulfonic acid group (–SO_3_H) on the aromatic ring which is connected to the hydrophilic alkyl chain that makes it soluble in alcohol, whilst the aromatic ring is hydrophobic which makes it soluble in oil. So DBSA catalyst improves the solubility of alcohol and oil due to their molecular structure. Therefore, the rate of reaction also improves due to increased mass transfer rates between alcohol and oil phases due to improved solubility.

Investigations have proved that with the DBSA catalyst, both the transesterification and esterification reactions occur efficiently using conventional heating method^16,17^. But high conversion for transesterification reaction could be achieved in about 6 h of reaction time which is still quite large^16^. Higher conversions for shorter reaction times can only be achieved at either high reaction temperatures (approx. 90 °C), or higher alcohol and catalyst quantity (methanol-to-oil molar ratio of 6:1 to 9:1 and the catalyst-to-oil molar ratio of 0.09:1 to 0.11:1)^16,17^. So the aim of the present investigation was to reduce this reaction time of transesterification of triglycerides further from 6 h using DBSA catalyst to achieve more than 95% conversion under mild operating conditions.

These days the microwave technique has attracted significant attention because of its ability to complete reactions in extremely short times. Microwaves are electromagnetic radiations that are capable to heat polar molecules of the reactants that tend to arrange themself with the electromagnetic field and generate heat by the friction of the molecules. Another mechanism is a non-thermal effect of the microwave which is related to the uncoupling of the spin of electrons present in the atoms that leads to faster reaction mechanisms^18,19^. So, many researchers around the world have recommended it for organic and inorganic syntheses^20^. Till date, lots of studies have been carried out to produce biodiesel by transesterification using microwave methods^21–35^. Some of the studies were also on the production of biodiesel by high FFA oil using acid catalysts by microwave heating. Lukasz et al.^29^ compared both conventional heating and microwave heating methods for the production of biodiesel by transesterification process with vegetable oil in the presence of a variety of solid acidic catalysts, p-toluenesulfonic acid and Nafion NR 50 and liquid sulfuric acid. Both the heating methods gave an excellent yield of more than 85%, however with microwave heating the reaction was completed in shorter reaction times; 1 h compared to more than 24 h with conventional heating. Another study was carried out by Lokman et al.^36^ on microwave-assisted esterification of palm fatty acid distillate, using a sulfonated-glucose solid acid catalyst. The results of this study revealed the potential of microwave irradiation that enhanced the reaction rate by eight folds, improved the yield, and reduced the production cost compared to the conventional heating method under mild operating conditions. Leadbeater et al. performed experiments, intending to check the effect of microwave heating on the reaction rate of the acid-catalyzed route^37^. Biodiesel was produced with 93% conversion by transesterification of vegetable oil under microwave heating using methanol to oil molar ratio of 30:1, 5wt% H_2_SO_4_, at 150 °C for 10 min of reaction time in a sealed vessel. With butanol, a similar conversion was achieved with oil to butanol ratio of 1:6, 5wt% H_2_SO_4_, at 120 °C in only 2 min of reaction time when performed in an open vessel. Three acidic imidazolium ionic liquids were synthesized by Ding et al.^38^, for the production of biodiesel from palm oil using microwave irradiations by a single step process. Biodiesel yield of 98.93% was obtained with11:1 methanol to oil molar ratio, 9.17% ionic liquid dosage, under 168 W microwave power in 6.43 h of reaction time. The above-mentioned studies reflected the positive effect of microwave heating to enhance the rate of reaction of transesterification/esterification in the presence of an acid catalyst. There are a large number of studies published in the literature about the production of biodiesel by single-step transesterification using high FFA oils by applying microwave heating in the presence of acid catalysts. However, the literature on the use of microwave methods for transesterification of triglycerides using organic acid catalysts, specifically DBSA, for improving the rate of reaction is hard to find. Therefore, an attempt was made in this work to fill this gap. To optimize the reaction variables for the higher conversion of biodiesel, the central composite design (CCD) matrix of response surface methodology (RSM) using Design Expert 11 software was applied for the design of the experiment. To find out the best model of the process, various reduced regression models were compared with the help of ANOVA, main effect plots, interaction plots, surface plots, and contour plots and finally, a modified polynomial model was developed to predict the conversion of triglycerides by the transesterification process using DBSA catalyst by microwave heating for the production of fatty acid methyl esters (FAME). The model was also validated by performing additional experiments using the optimum values of variables.

## Experimental method

### Chemicals and equipment

Refined sun flower oil (as a source of pure triglycerides) and methanol (> 99.8% pure, Sigma-Aldrich) were used as feedstocks for the transesterification process. The catalyst was DBSA (a mixture of isomers, ≥ 95%, Sigma-Aldrich). All reagents were used as purchased, without any purification. The microwave reactor used was the Flexi Wave Model of Milestone Company, Italy, fitted with a magnetic stirrer for continuous stirring and an infrared temperature sensor, which enabled and controlled the temperature.

### Microwave heating assisted transesterification

Transesterification reactions with pure triglycerides and methanol in the presence of DBSA catalyst were performed by microwave heating to see its effect on the rate of reaction of the process. The reaction conditions for all the experimental runs are shown in Table 2. For every experimental run, the pre-specified oil and catalyst molar ratios were added in a 100-mL sealed reactor (a Teflon vessel), which was placed inside the microwave cavity to ensure the reaction mixture would attain the preset operating temperature. The pre-calculated volume of the preheated methanol was then mixed into the reactor. This reaction mixture was then irradiated using the power of 300 W and agitated at around 300 rpm by magnetic stirring. The advancement of the reaction was determined at various reaction times (10, 30, 60, and 120 min.) by analyzing the quantity of un-reacted triglycerides and FAME (biodiesel) formed in the reaction mixture by proton nuclear magnetic resonance spectroscopy (^1^H NMR). Each run was conducted at least two or three times for the reproducibility of the results.

### Purification of biodiesel

The transesterification reaction was terminated by placing the reaction vessel in cold water immediately after it was taken out from the reactor. Microwave irradiation can significantly help in achieving a good phase separation. The reaction product was then kept in a separating funnel for a few minutes. The glycerol layer present at the lower part of the funnel was separated from the upper biodiesel containing layer which was further purified. The collected layer was pressured washed with sparger of warm water for four to five times until the washed water was neutral to litmus test and finally dried by heating at 100 °C. The acidity of purified biodiesel was then checked by the standard procedure (ASTM D974) and was in agreement with ASTMD6751. The removal of the catalyst was also confirmed by the ^1^H NMR spectrum of the sample which showed no catalyst peak after purification (Fig. S16 of supplementary file).

### Analysis using ^1^H NMR spectroscopy

**Figure 1:**
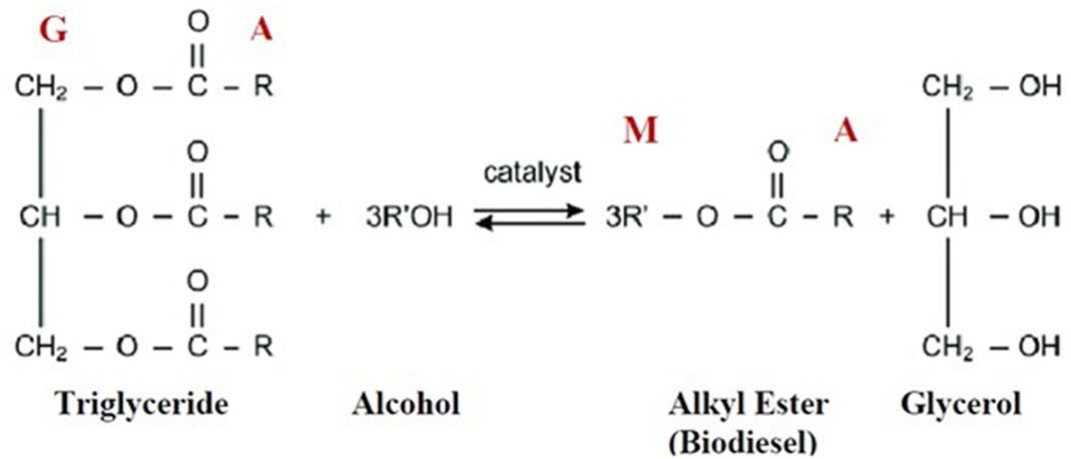
Transesterification reaction with highlighted protons.

The proton nuclear magnetic resonance spectroscopy (^1^H NMR) technique was used for quantitative analysis. The amplitude of a ^1^H NMR signal is dependent on the number of hydrogen in the molecule^39,40^. Even though HPLC and GC are more sensitive methods in comparison to NMR, the latter is a more quick and simple technique than the former^41,42^. All the reaction samples were analyzed by Bruker Avance 400 (FT NMR) using chloroform (CDCl_3_) as a solvent. Figure 1 shows the transesterification reaction of triglycerides, in which the protons used to monitor the reaction are bold and represented by the letters M (methyl ester), A (α-CH_2_ ions), and G (glyceridic).

The related signals selected were of the protons of the methyl ester moiety (M) associated with the FAME at 3.67 ppm (3H) and of methylene groups (G) of the glyceridic protons at 4.07–4.35 ppm (4H). Though there were a total five glyceridic protons, the signal for the glyceryl methine lay at 5.27 ppm. Therefore Eq. (1) was used to calculate the conversion of triglycerides to biodiesel (X), where I_TG_ and I_ME_ give the areas under the curves of the glyceridic protons and the methyl ester protons, respectively^16,43^.

1 $$X(\% ) = \frac{{4I_{ME} }}{{4I_{ME} + 9I_{TG} }} \times 100$$

In the above equation, the factor of four represents the four glyceryl methylenic hydrogens present in the triglyceride molecule. The factor of nine signifies the hydrogen atoms formed in three ethyl ester products. The sample graphs of the ^1^H NMR spectrum of biodiesel can be seen in Fig. S16 of the supplementary file.

### Central composite design (CCD) matrix

To optimize the process conditions for higher conversions, the response surface methodology (RSM) was used. It was reported^16^ that a highly significant curvature effect in the regression model developed for the conventional heating transesterification process for biodiesel production using the DBSA catalyst was observed. Therefore, a CCD was selected in this study as well to explore the relation between response and factors. Design Expert 11 software was selected for optimizing the level of the variables. A three-variable CCD was used and 20 experiments were conducted to optimize the three independent variables, which were reaction temperature (A), the catalyst to oil molar ratio (B), and methanol to oil molar ratio (C). The actual levels of the three independent variables in un-coded and coded values are shown in Table 1.

**Table 1:** Factors and levels for transesterification process.

Factors	Levels				
	− Alpha(− 1.682)	− 1	0	+ 1	+ Alpha(+ 1.682)
Reaction temperature (A),°C	60	64	70	76	80
Catalyst to oil molar ratio (B)	0.0095	0.03	0.06	0.09	0.11
Methanol to oil molar ratio (C)	0.95	3.0	6.0	9.0	11.0

The low and high levels of temperature were selected as 64 and 76 °C, respectively. The range of temperature was selected such that it should not be much higher from the boiling point of alcohol to avoid the excess rise of pressure in the sealed Teflon vessel. This range of temperature was higher than that used for conventional alkaline catalyzed reaction, but the difference was almost insignificant, and this higher temperature made purification of product mixture easy^16^.

The selected levels of catalyst to oil molar ratios were similar to the earlier works with acid organocatalysts^16^. The amount of catalyst should be as low as possible to minimize the operational cost but should be sufficient to get a high yield of biodiesel (> 95%).

The low and high levels of alcohol to oil molar ratios were taken as 3:1 and 9:1, respectively. These levels were low as compared to the previous works on heterogeneous or homogeneous inorganic acidic catalysts, in which molar ratios of alcohol up to 30:1 were common^4,44^. The methanol amount was reduced owing to increased solubility of the two phases of oil and methanol due to the molecular structure of the catalyst DBSA^2^.

The selected design contained 8 factorial points, 6 axial points, and 6 replicated center points (Table 2). The regression analysis was carried out to calculate the response function X as a second-order polynomial equation as follows:

2 $$X = {\upbeta }o + \sum\limits_{{(i = 1)}}^{n} {{\upbeta }_{i} {\text{x}}_{i} } + \sum\limits_{{(i = 1)}}^{n} {{\upbeta }_{{ii}} {\text{x}}_{i} ^{2} } + \sum\limits_{{(i = 1)}}^{n} {\sum\limits_{{j = 1}}^{{i - 1}} {{\upbeta }_{{ij}} x_{i} x_{j} + \varepsilon _{i} } }$$

where X in Eq. (2) is the predicted response, β_i,_ β_ii,_ and β_ij_ are the coefficients which represent the linear, quadratic, and interaction effects of x_1_, x_2_, x_3,_ etc., whereas n represents the number of independent parameters and ε is the random error^45^. Design Expert 11 software was used to carry out the design, analysis, model fitting, and graph plotting.

**Table 2:** Different factors, levels, and values of the responses.

Standard order	Run order	Levels of factor						Values of response			
		Reaction temperature		Catalyst to oil molar ratio		Methanol to oil molar ratio		Conversion of triglyceride into biodiesel (%)			
		Real (°C)	Coded	Real (molar ratio)	Coded	Real (molar ratio)	Coded	10 min	30 min	60 min	120 min
16	1	70.00	0	0.060	0	6.00	0	21.66	49.79	66.59	70.70
10	2	80.00	+ 1.682	0.060	0	6.00	0	53.61	83.10	> 99.50	> 99.50
14	3	70.00	0	0.060	0	11.00	+ 1.682	33.46	57.21	73.78	> 99.50
8	4	76.00	+ 1	0.090	+ 1	9.00	+ 1	66.16	> 99.50	> 99.50	> 99.50
17	5	70.00	0	0.060	0	6.00	0	22.94	48.24	68.38	72.61
19	6	70.00	0	0.060	0	6.00	0	21.87	47.62	67.46	71.39
4	7	76.00	+ 1	0.090	+ 1	3.00	− 1	26.23	47.22	73.63	> 99.50
5	8	64.00	− 1	0.030	− 1	9.00	+ 1	17.47	37.91	50.53	68.57
11	9	70.00	0	0.009	− 1.682	6.00	0	10.19	18.18	33.75	42.79
15	10	70.00	0	0.060	0	6.00	0	23.05	47.56	68.55	73.04
1	11	64.00	− 1	0.030	− 1	3.00	− 1	09.43	17.82	34.62	57.14
13	12	70.00	0	0.060	0	0.95	− 1.682	15.72	30.64	35.71	37.21
3	13	64.00	− 1	0.090	+ 1	3.00	− 1	20.52	46.00	59.46	65.49
2	14	76.00	+ 1	0.030	− 1	3.00	− 1	14.87	29.00	55.85	65.23
18	15	70.00	0	0.060	0	6.00	0	21.94	49.35	66.21	70.85
9	16	60.00	− 1.682	0.060	0	6.00	0	12.64	25.25	39.91	59.59
6	17	76.00	+ 1	0.030	− 1	9.00	+ 1	24.21	58.36	71.89	81.53
7	18	64.00	− 1	0.090	+ 1	9.00	+ 1	46.61	67.15	69.51	74.15
20	19	70.00	0	0.060	0	6.00	0	22.66	48.54	66.49	70.53
12	20	70.00	0	0.110	+ 1.682	6.00	0	39.76	66.87	> 99.50	> 99.50

## Results and discussion

### Analysis of variance (ANOVA) and the main effects of factors

The different levels of the factors and their responses by a rotatable central composite design are shown in Table 2. To study the relation of factors on their responses, the conversions were determined at four different reaction times (10, 30, 60, and 120 min.).

ANOVA result for 60 min of reaction is shown in Table 3 (ANOVA results for 10, 30, and 120 min. are provided in the supplementary file as Tables S1, S2, and S3, respectively). In an initial attempt, the model including all quadratic and two-factor interaction terms apart from main effects was fitted to experiments of 60 min of time by the multiple linear regression method. The response for 60 min of time was correlated with the three selected variables using the following polynomial Eq. (3)

3 $${\text{X}}_{(60\min .)} (\% ) = 67.25 + 13.79{\text{A}} + 14.73{\text{B}} + 9.69{\text{C}} + 0.26{\text{AB}} + 2.06{\text{AC}} + 0.56{\text{BC}} + 1.11{\text{A}}^{2} + 0.02{\text{B}}^{2} - 4.27{\text{C}}^{2}$$

where X is the response variable, that is, conversion of triglyceride into FAME, whereas A, B, and C are the real values of the predictor variables namely, reaction temperature, the molar ratio of catalyst to oil, and the molar ratio of alcohol to oil, respectively. The linear, quadratic, or interaction effects on the response were checked for significance by ANOVA which is presented in Table 3 for 60 min response. The large value of the coefficient of multiple determinations for R^2^ = 0.9393 and adjusted R^2^ = 0.8846 indicated good fitness of the results and adequately presented the experimental results. However, the predicted R^2^ of 0.5361 was quite different from the adjusted R^2^ of 0.8846, i.e., the difference was more than 0.2. This may have signified a possible problem with the model. F-test at 95% confidence level (α = 0.05) showed that the regression model was significant and adequate. Residual error (1 – R^2^) was less than 10% except for the model for 120 min where the residual error was 15%. The main effects of reaction temperature (A), the molar ratio of catalyst to oil (B), and the molar ratio of methanol to oil (C) on the conversion were significant as p-values were less than 0.05. Beyond 10 min (initial phase of the reaction), all the two-factor interactions and quadratic effects were insignificant (*p*-value > 0.05) except the *p*-value of the quadratic effect of the molar ratio of methanol to oil (C^2^) was 0.0388 (< 0.05) for 60 min of reaction. However, p-values of the main effects of all the factors were in the order of 10^–3^ or 10^–4^. This meant that within the range of the factors analyzed, only the main effects of temperature (A), the molar ratio of catalyst to oil (B), and the molar ratio of methanol to oil (C) on the conversion would dominate throughout the reaction.

**Table 3:** ANOVA for the quadratic polynomial model for 60 min of reaction time.

Source	Sum of squares	DF	Mean Square	F-value	*P*-value	
**Model**	7177.24	9	797.47	17.19	< 0.0001	Significant
A-Reaction temperature	2596.69	1	2596.69	55.97	< 0.0001	Significant
B-Catalyst to oil molar ratio	2962.37	1	2962.37	63.85	< 0.0001	Significant
C-Methanol to oil molar ratio	1283.38	1	1283.38	27.66	0.0004	Significant
AB	0.53	1	0.53	0.011	0.917	
AC	33.80	1	33.80	0.729	0.413	
BC	2.50	1	2.50	0.054	0.821	
A^2^	17.79	1	17.79	0.383	0.549	
B^2^	0.0075	1	0.0075	0.0002	0.990	
C^2^	262.29	1	262.29	5.65	0.039	
**Residual**	463.97	10	46.40			
Lack of fit	458.89	5	91.78	90.41	< 0.0001	Significant
Pure error	5.08	5	1.02			
**Cor total**	7641.21	19				

R^2^ = 0.9393.

Adjusted R^2^ = 0.8846.

Predicted R^2^ = 0.5361.

**Figure 2:**
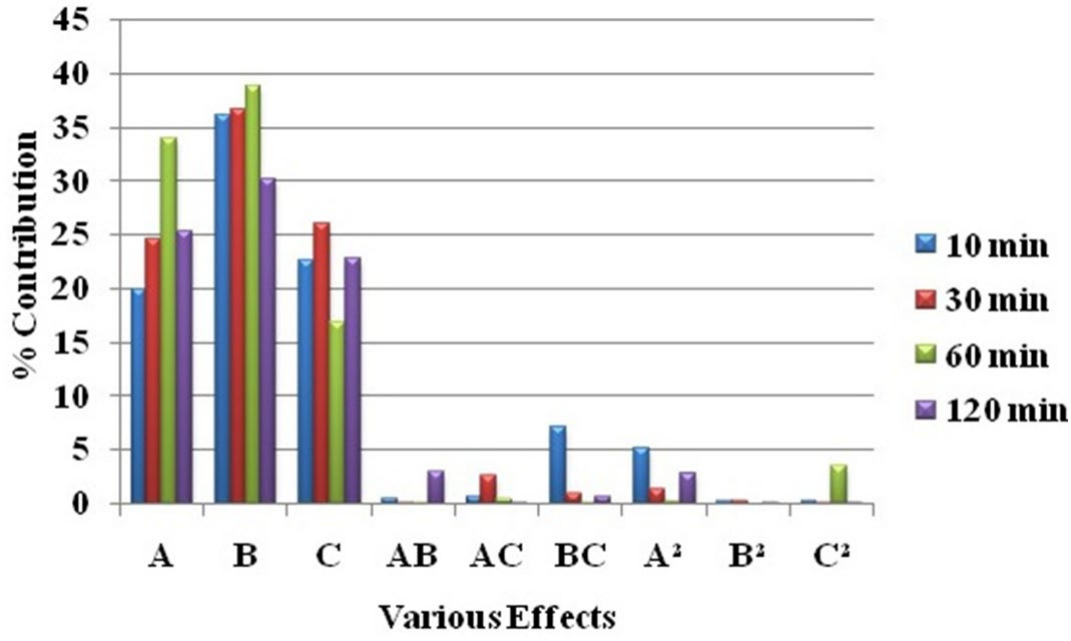
Relative contribution of the various effects.

A relative contribution of the various effects were calculated from ANOVA (Table 4) as a ratio of the sum of the square of the effect to the total sum of squares and plotted in Fig. 2 which depicts that the main effects of temperature (A), the molar ratio of catalyst to oil (B), and the molar ratio of methanol to oil (C) on the conversion were dominating throughout the reaction. The relative contribution of each main effect increased up to 60 min and then decreased. This was obvious because as the reaction approached higher conversion, the effect of parametric variations on the conversion would reduce after some time. The relative trends for the main effects were found to be in the order: molar ratio of catalyst to oil (B) > molar ratio of methanol to oil (C) > reaction temperature (A) up to 60 min and the molar ratio of catalyst to oil (B) > temperature (A) > molar ratio of methanol to oil (C) beyond 60 min. The molar ratio of catalyst to oil (B) was the most dominating among all of the factors throughout the reaction. Alegria et al.^16^ also reported catalyst DBSA to oil molar ratio (B) as the most dominating factor and the relative trend of the main effects in the order: molar ratio of catalyst to oil (B) > temperature (A) > molar ratio of methanol to oil (C) after 3 h of reaction.

**Table 4:** Relative contribution of the various effects**.**

Factors	Percent contribution of factors			
	For 10 min. of reaction time	For 30 min. of reaction time	For 60 min. of reaction time	For 120 min. of reaction time
A	19.89	24.53	33.98	25.22
B	36.09	36.62	38.77	30.17
C	22.55	26.08	16.80	22.79
AB	0.51	0.01	0.01	2.98
AC	0.69	2.64	0.44	0.03
BC	7.11	0.95	0.03	0.70
A^2^	5.19	1.40	0.23	2.87
B^2^	0.34	0.33	0.00	0.09
C^2^	0.25	0.13	3.43	0.03
**Sum of main effects**	78.53	87.23	89.55	78.19

**Figure 3:**
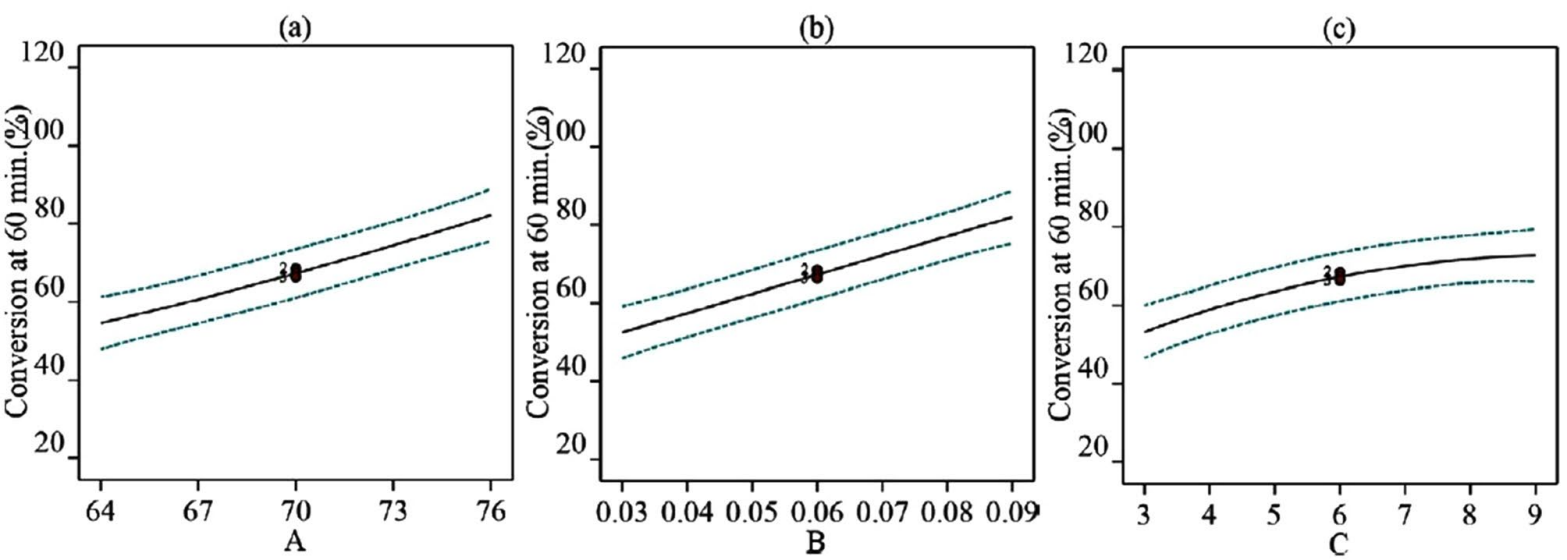
Main effects of factors on the conversion of triglyceride to biodiesel at 60 min of reaction time (**a**) Effect of reaction temperature (A) [At B = 0.06 and C = 6] (**b**) Effect of the molar ratio of catalyst to oil (B) [At A = 70 °C and C = 6] (**c**) Effect of the molar ratio of methanol to oil (C) [A = 70 °C and B = 0.06].

Plots for the main effects of reaction temperature (A), the molar ratio of catalyst to oil (B), and the molar ratio of methanol to oil (C) are shown in Fig. 3 for 60 min of reaction (Fig. S1, S2, and S3 in supplementary file material are for 10, 30, and 120 min, respectively). Conversion of triglycerides to biodiesel increased with temperature (A), the molar ratio of catalyst to oil (B), and the molar ratio of methanol to oil (C) which meant that all the factors have positive main effects on the conversion throughout the reaction.

### Contour plots and response surfaces

**Figure 4:**
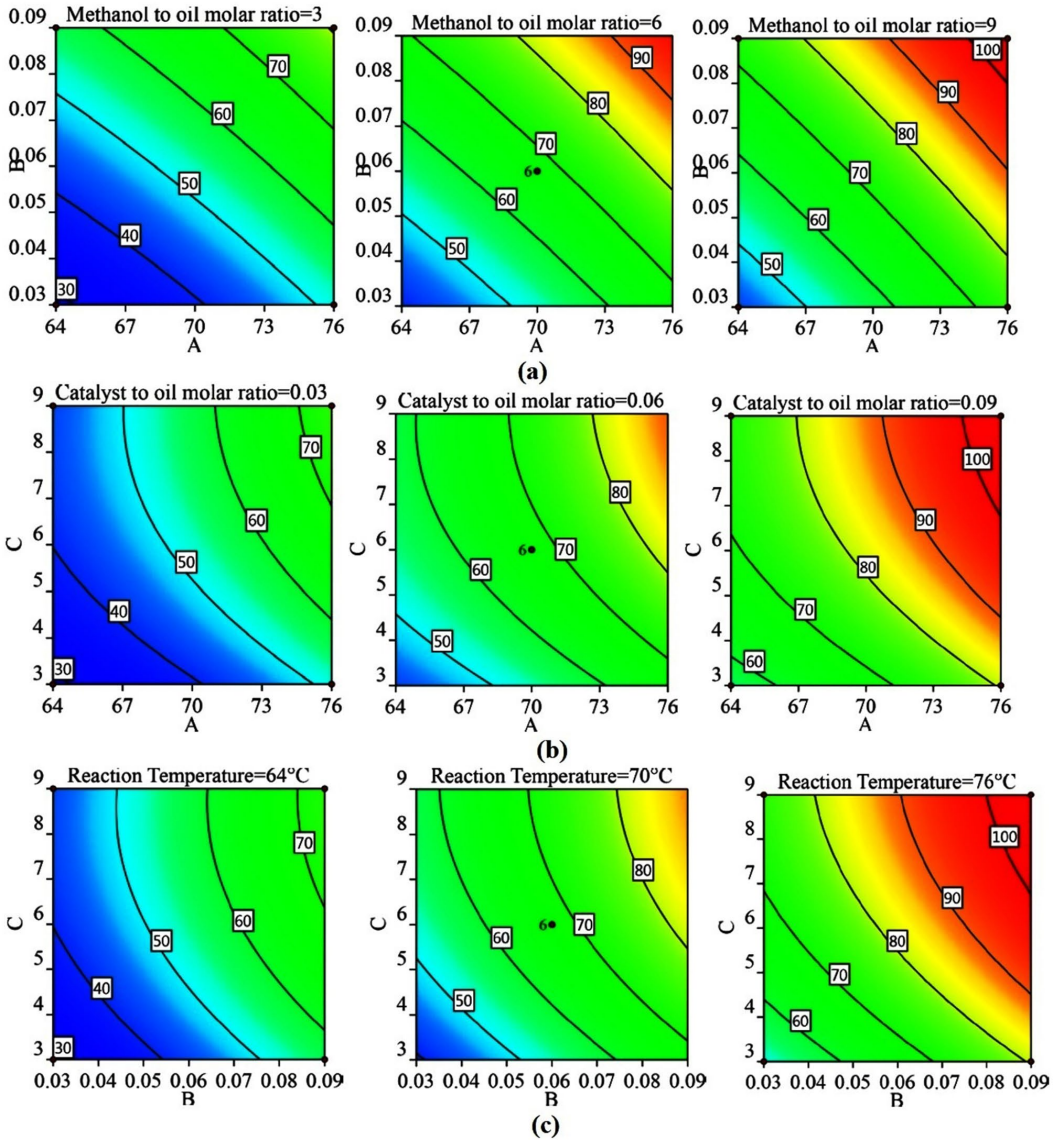
Contour plots at 60 min. of reaction at lower (− 1), middle (0) and upper limits (+ 1) of fixed factors (**a**) Reaction temperature − molar ratio of catalyst to oil, (**b**) Reaction temperature − molar ratio of methanol to oil, (**c**) Molar ratio of catalyst to oil − molar ratio of methanol to oil.

**Figure 5:**
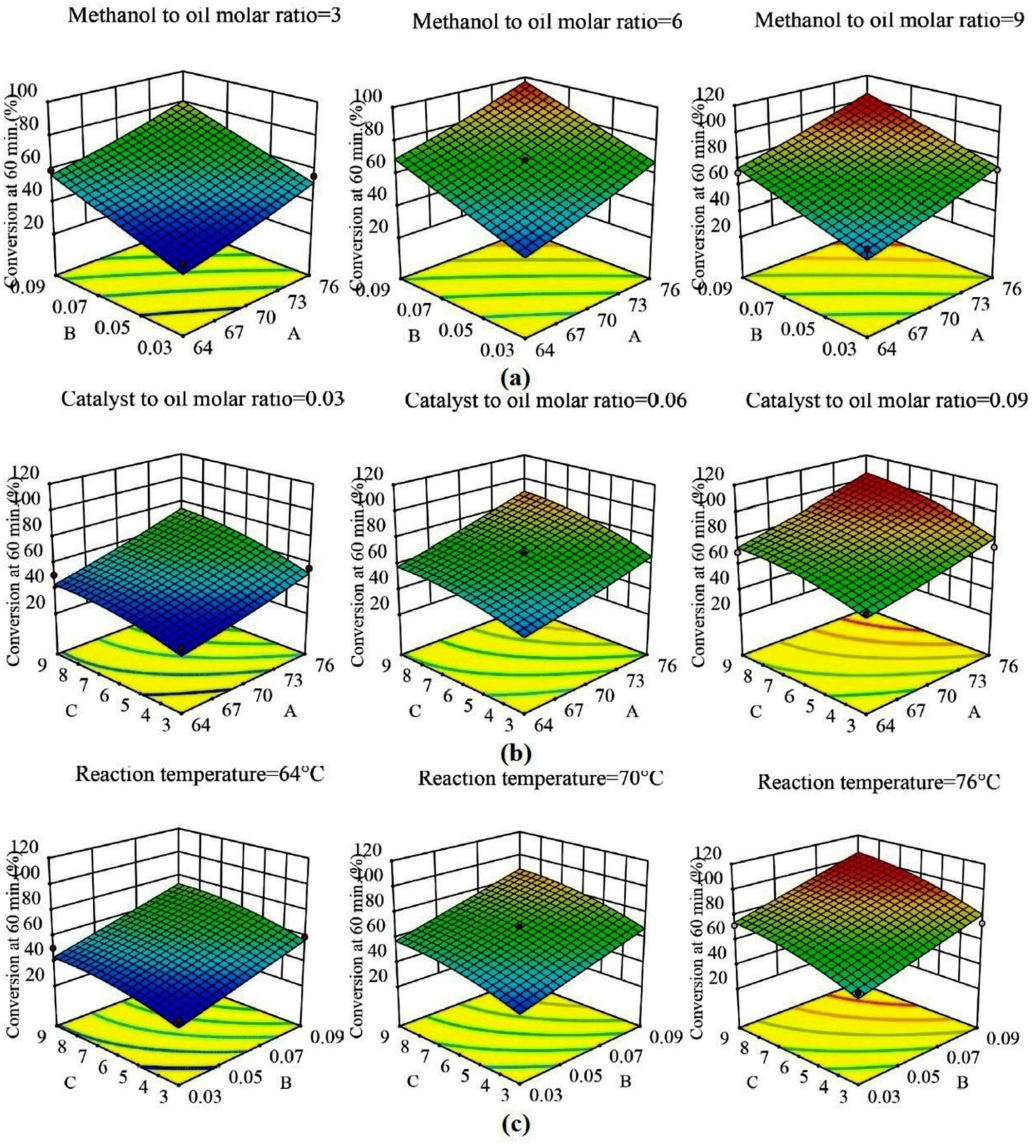
Response surface plots for 60 min of reaction at lower (− 1), middle(0) and upper limits (+ 1) of fixed factors (**a**) Reaction temperature (A) − molar ratio of catalyst to oil (B), (**b**) Reaction temperature (A) − molar ratio of methanol to oil (C), (**c**) Molar ratio of catalyst to oil (B) − molar ratio of methanol to oil (C).

Contour plots and response surfaces for 60 min of reaction are shown in Figs. 4 and 5, respectively (the contour plots in Fig. S4, S5, and S6 and response surfaces in Fig. S7, S8, and S9 for 10, 30, and 120 min, are shown respectively in the supplementary file). Nature of all the contour plots and response surfaces implied the non-existence of maxima, minima, or saddle point in the process within the range of factors selected. All the contour plots and response surfaces showed that there was a monotonous, but non-linear, increase in the conversion of triglycerides to biodiesel by an increase in each factor. The non-linearity was mainly contributed by the following: two-factor interactions and curvature effects (quadratic terms). The relative contribution of all the main effects was ranging from 78.53 to 89.55% (Table 4) and beyond 10 min (initial phase of the reaction), the relative contribution of the remaining effects (excluding residual error) was found to be less than 7%. Therefore, the causes of the non-linearity were required to be explored.

**Figure 6:**
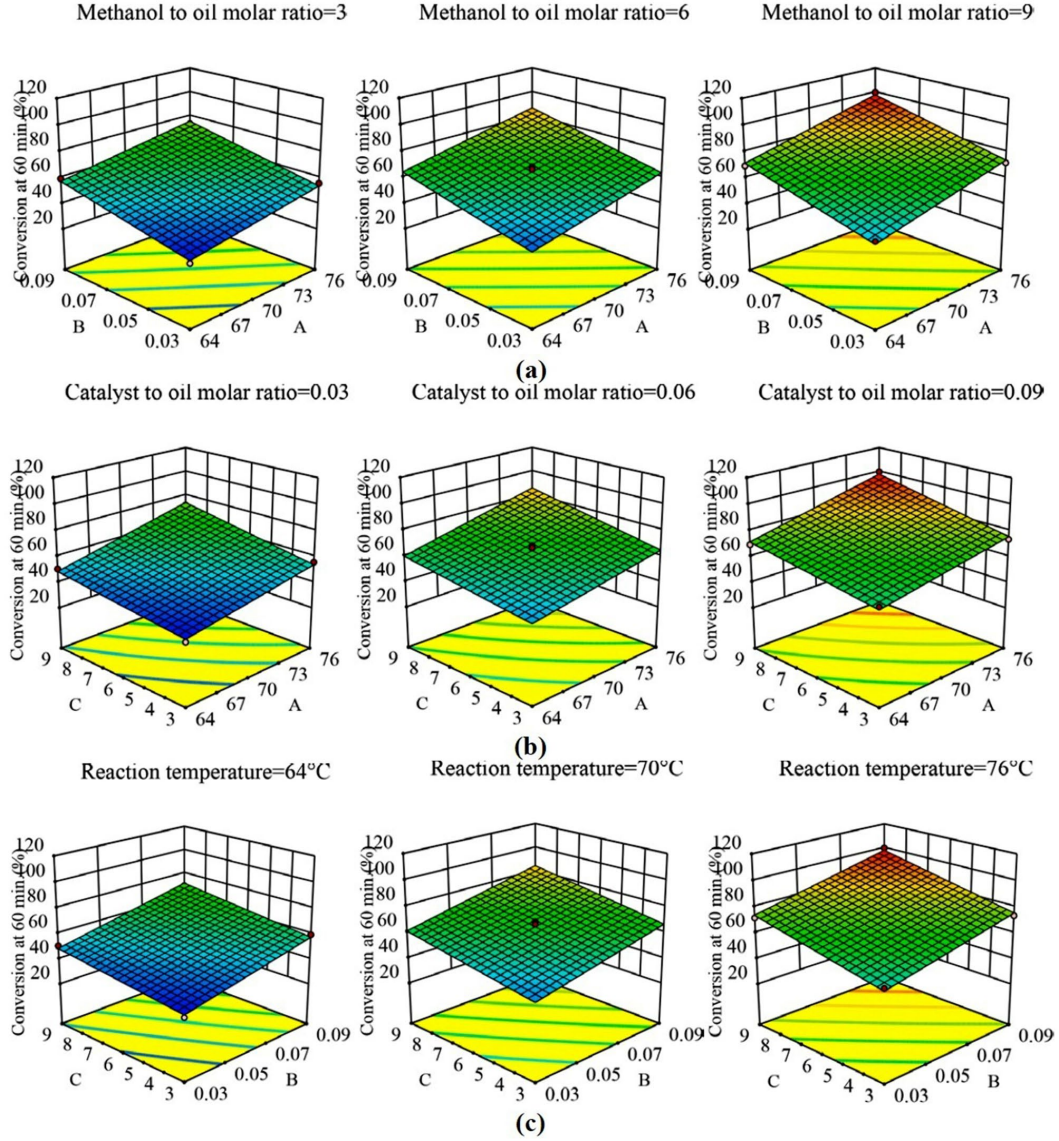
Surface plots of factorial design for 60 min of reaction time at lower (− 1), middle (0) and upper limits (+ 1) of fixed factors (**a**) Reaction temperature (A) − molar ratio of catalyst to oil (B), (**b**) Reaction temperature (A) − molar ratio of methanol to oil (C), (**c**) Molar ratio of catalyst to oil (B) − molar ratio of methanol to oil (C).

**Figure 7:**
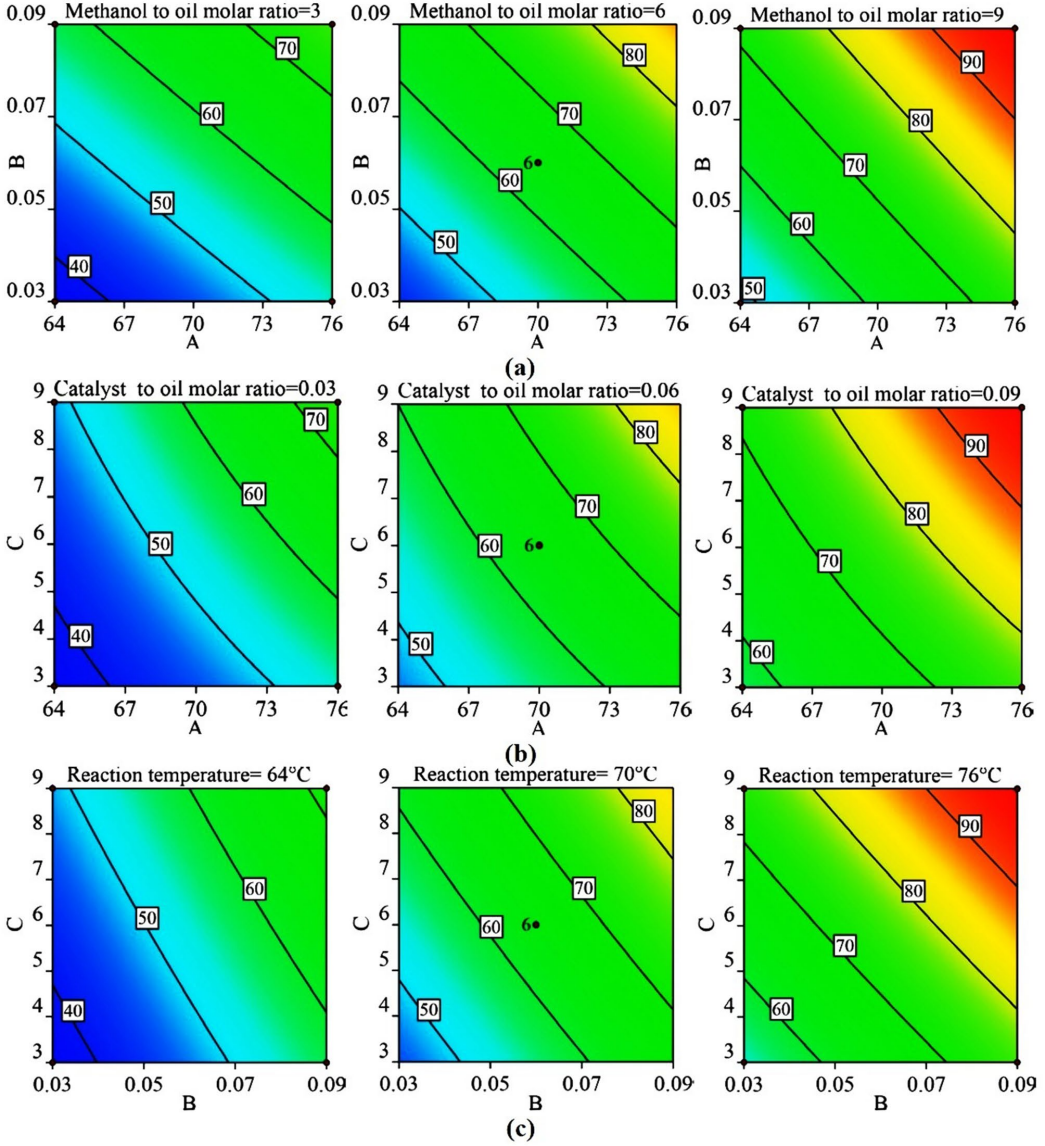
Contour plots of factorial design at 60 min. of reaction time at lower (− 1), middle (0) and upper limits (+ 1) of fixed factors (**a**) Reaction temperature − molar ratio of catalyst to oil, (**b**) Reaction temperature − molar ratio of methanol to oil, (**c**) Molar ratio of catalyst to oil − molar ratio of methanol to oil.

A two-level factorial design with center points was used to explore the curvature effects. All the eight factorial points and six center points from the CCD design in Table 5 were used to determine the curvature effects. In this analysis, the regression model consisted of terms for the main effects and the two-parameter interaction effects, and consequently Eq. (3) was modified. ANOVA results and surface plots from this analysis are provided in Table 6 and Fig. 6 for 60 min of reaction, respectively (see supplementary file for 10, 30, and 120 min). Though the ANOVA results showed that the overall effect of curvature was insignificant beyond 10 min (initial phase) of the reaction, the response surface was not passing through the center points (i.e., center points were either above or below the response surface), whereas the full quadratic model (Eq. 3) generated response surfaces in Fig. 7 were passing through the center points. Therefore, though the overall effect of curvature was insignificant, selective quadratic effect(s) could be considered in the model to achieve better fitting. It was also evident from the plots in Fig. S1, S2, and S3 that the main effects of the factors were almost linear throughout the reaction except for a little curvature (or, non-linearity) in the main effect of temperature (A) at the 10 min of reaction and methanol to oil molar ratio (C) at 60 min of reaction. ANOVA results also supported the presence of a quadratic effect of temperature (A^2^) at 10 min and methanol to oil molar ratio (C^2^) at 60 min. In the analysis of factorial design with center points (discussed in the preceding paragraph), the non-linearity in the contour plots (Fig. 7) and response surfaces (Fig. 6) appeared even though no quadratic effects were present in the model. Furthermore, ANOVA results for the full quadratic model inferred that the overall effects of two-parameter interactions were insignificant. But slight non-linearity in the contour plots and response surfaces suggested further investigation of two-factor interactions.

**Table 5:** Two-level factorial design with centre points and values of the responses at various reaction times.

Std order	Run order	A: Reaction Temperature (**°**C)	B: Molar ratio of catalyst to oil	C: Molar ratio of methanol to oil	Conversion at 10 min (%)	Conversion at 30 min (%)	Conversion at 60 min (%)	Conversion at 120 min (%)
8	1	76	0.09	9	66.16	> 99.50	> 99.50	> 99.50
12	2	70	0.06	6	21.94	49.35	66.21	70.85
6	3	76	0.03	9	24.21	58.36	71.89	81.53
7	4	64	0.09	9	46.61	67.15	69.51	74.15
13	5	70	0.06	6	21.87	47.62	67.46	71.39
10	6	70	0.06	6	21.66	49.79	66.59	70.70
9	7	70	0.06	6	23.05	47.56	68.55	73.04
2	8	76	0.03	3	14.87	29.0	55.85	65.23
3	9	64	0.09	3	20.52	46.00	59.46	65.49
4	10	76	0.09	3	26.23	47.22	73.63	> 99.50
5	11	64	0.03	9	17.47	37.91	50.53	68.57
1	12	64	0.03	3	9.43	17.82	34.62	57.14
11	13	70	0.06	6	22.94	48.24	68.38	72.61
14	14	70	0.06	6	22.66	48.54	66.49	70.53

**Table 6:** ANOVA for the factorial model of 60 min of reaction time.

Source	Sum of squares	DF	Mean square	F-value	*P*-value	
**Model**	2579.18	6	429.86	68.18	< 0.0001	Significant
A-Reaction temperature	951.64	1	951.64	150.94	< 0.0001	Significant
B-Catalyst to oil molar ratio	1006.28	1	1006.28	159.61	< 0.0001	Significant
C-Methanol to oil molar ratio	584.43	1	584.43	92.70	< 0.0001	Significant
AB	0.53	1	0.53	0.0841	0.782	
AC	33.80	1	33.80	5.36	0.06	
BC	2.50	1	2.50	0.3970	0.552	
Curvature	27.75	1	27.75	4.40	0.081	
**Residual**	37.83	6	6.30			
Lack of fit	32.75	1	32.75	32.26	0.0024	Significant
Pure error	5.08	5	1.02			
**Cor total**	2644.75	13				

R^2^ = 0.9855.

Adjusted R^2^ = 0.9711.

Predicted R^2^ = 0.1962.

### Interaction plots

**Figure 8:**
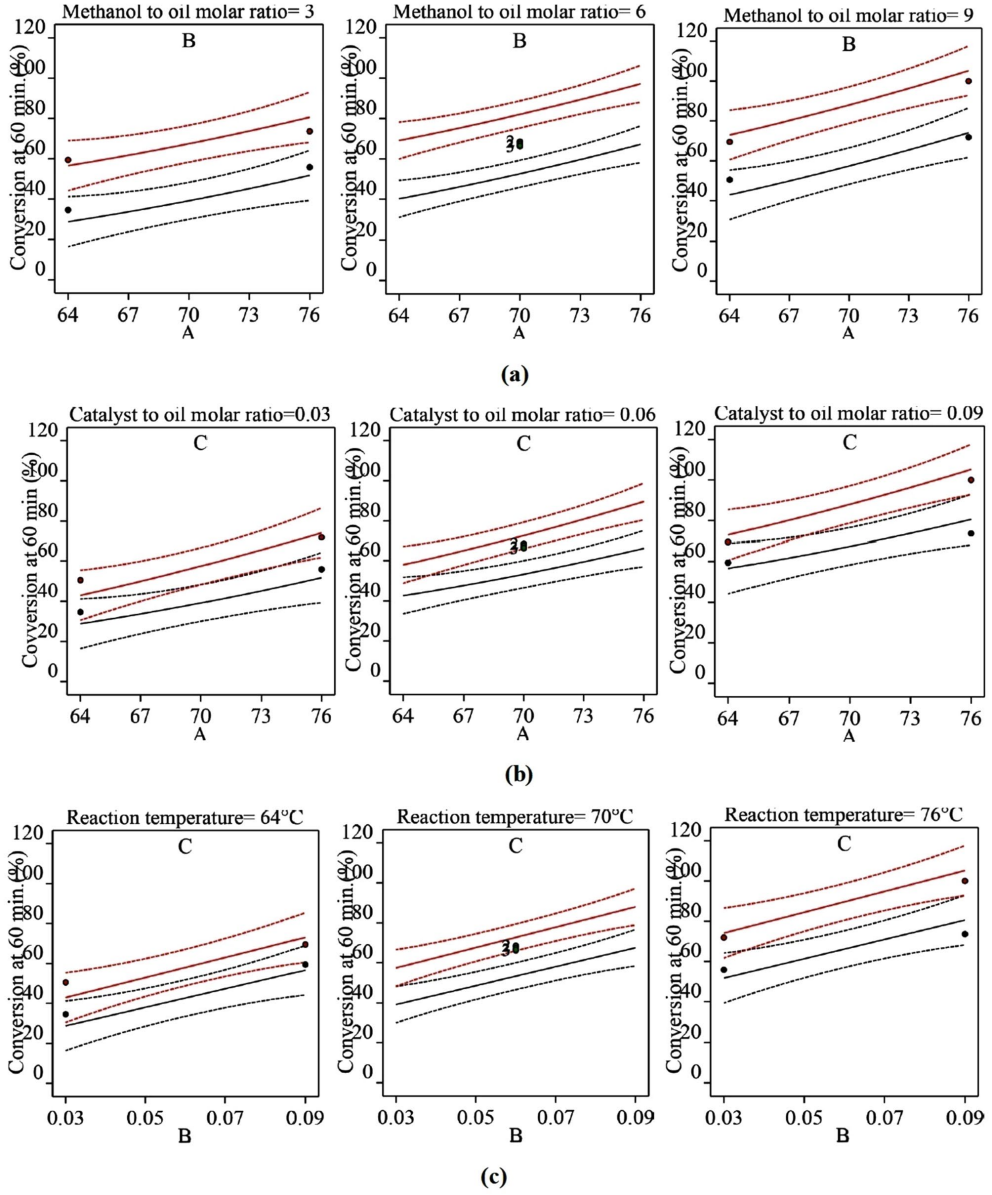
Interaction effects of factors on the conversion of triglyceride to biodiesel at 60 min of reaction time (**a**) Reaction temperature − molar ratio of catalyst to oil (B − 0.03, B + 0.09), (**b**) Reaction temperature − molar ratio of methanol to oil (C − 3, C + 9), (**c**) Molar ratio of catalyst to oil − molar ratio of methanol to oil (C − 3, C + 9).

Interaction plots were generated as shown in Fig. 8 for 60 min of reaction (see supplementary file S13, S14, and S15, respectively, for 10, 30, and 120 min) to understand whether the non-linearity was caused by two-factor interactions. There are usually three types of interactions: independent, synergistic, and anti-synergistic. Independent means no-interaction and lines drawn between parameters will be parallel to each other, irrespective of non-linearity or linearity. Synergistic means the effect of the two-parameter interaction results in a more positive effect on the response than the collective main effects of individual parameters, and anti-synergistic means effect of two-parameter interaction results in less impact on the response than the collective main effects of individual parameters.

From the interaction plots in Fig. 8 (see supplementary file for 10, 30, and 120 min), it was seen that the two-factor interactions were either independent or slightly synergistic in the present study. Most of the effects due to the two-factor interactions were synergistic. At 60 min (Fig. 8a), the temperature and catalyst to oil molar ratio interaction (AB) were independent as both the solid lines were parallel, because of the same increase in conversion (i.e., delta X ≈ 30%) at 64 °C (from 40 to 69%) and at 76 °C (from 67 to 97%) while increasing molar ratio of catalyst to oil (B) from 0.03 to 0.09 for methanol to oil molar ratio of 6. Similarly, the molar ratio of catalyst to oil and molar ratio of methanol to oil interaction (BC) was independent because the same increase in conversion (i.e., delta X ≈ 19%) at 0.03 catalyst to oil molar ratio (B) (from 39% to 57.5%) and at 0.09 catalyst to oil molar ratio (68% to 88%) while increasing molar ratio of methanol to oil (C) from 3 to 9. Whereas, temperature and molar ratio of methanol to oil interaction (AC) was synergistic because increasing methanol to oil molar ratio (C) from 3 to 9 resulted in more conversion (from 66 to 90%, i.e., delta X = 24%) at 76 °C than the conversion obtained at 64 °C (from 43 to 58%, i.e., delta X = 15%). The synergistic effect of AC interaction might have resulted in slight non-linearity in the response surface. Therefore, the full quadratic regression model (Eq. 3) was modified, and hereafter all the models were called as reduced models.

### Model discrimination

Various reduced regression models were compared in Table 7 and a final reduced model was selected based on discriminating criteria such as R^2^, Adj. R^2^, Pred. R^2^, and outliers, where the outliers were defined as residual errors (Ɛ) > 5%.

**Table 7:** Comparison of various reduced models.

Model	Terms/effects in model	Std. Dev	R^2^	Adj.R^2^	Pred.R^2^	5% < Ɛ < 7%	7% < Ɛ < 10%	Ɛ > 10%	Total outliers
M1 (Full quadratic model)	A, B, C, AB, AC, BC, A^2^, B^2^, C^2^	6.81	0.9393	0.8846	0.5361	3	4	–	7
M2 (Main effects model)	A, B, C	7.07	0.8955	0.8759	0.8116	2	2	4	8
M3 (Main effects + 2FI term model)	A, B, C, AB, AC, BC	7.66	0.9003	0.8543	0.6528	3	2	3	8
M4 (Main effects + few 2FI term model )	A, B, C, AC	7.14	0.8999	0.8732	0.7958	4	2	3	9
M5 (Main effects + few 2FI + few quadratic term model)	A, B, C, AC, C^2^	5.89	0.9365	0.9139	0.8449	1	5	–	6
M6 (Main effects + few quadratic terms model)	A, B, C, C^2^	5.88	0.9321	0.914	0.8613	1	5	–	6

M1 (full quadratic model), in Table7, showed that the value of predicted R^2^ of 0.5361 was quite different from the adjusted R^2^ of 0.8846; i.e. the value of variation was more than 0.2. This may be an indicator of a probable problem with the model. There were 7 outliers that seem to have been the cause of the defect in the model. Whereas when interaction and quadratic terms were removed in model M2, the standard deviation and outlier increased from 6.81 to 7.07 and from 7 to 8, respectively, but predicted R^2^ of 0.8116 was reasonably close to adjusted R^2^ of 0.8759, i.e., the variation between them was less than 0.2. This showed that the model needed some interaction or quadratic terms. Model M3 that contained all the main effects and two-factor interaction (2FI) terms had the same outliers (8) but the standard deviation increased from 7.07 to 7.66 and the predicted R^2^ of 0.6528 was different from the adjusted R^2^ of 0.8543, which reflected a problem in the model. So the model had to be further modified as M4 which contained all the main effect and interaction effect terms of reaction temperature and the molar ratio of methanol to oil (AC) which showed curved lines on contour plots in Fig. 4b. Here although the standard deviation decreased from 7.66 to 7.14 but the number of outliers increased from 8 to 9. Thus, further modification in the model had to be done in the form of some appropriate quadratic term which could be C^2^ as it was a significant term in the full quadratic model (*p* = 0.0388). So the model M5 contained AC and C^2^ apart from all the main effects containing only 6 outliers for which the residual was not more than 10. The introduction of the C^2^ term showed remarkable improvement in the model. It could, therefore, be inferred that the non-linearity in the contour plots may have been due to the presence of C^2^. So in model M6, only the C^2^ term was taken together with the main effect terms and it showed the best results. The model M6 contained the lowest standard deviation of 5.88 and only 6 outliers. The predicted R^2^ of 0.8613 was reasonably equal to the adjusted R^2^ of 0.9140. This model could be used to find out the design space. So the final modified model (Eq. 4) for 60 min of reaction time was obtained as follows:

4 $${\text{X}}_{{({6}0 {\text{min}}.)}} \left( \% \right) = {68}.{1}0 + {13}.{\text{79A}} + {14}.{\text{73B}} + {9}.{\text{69C}} - {4}.{\text{37C}}^{{2}}$$

### Diagnostics of the model

**Figure 9:**
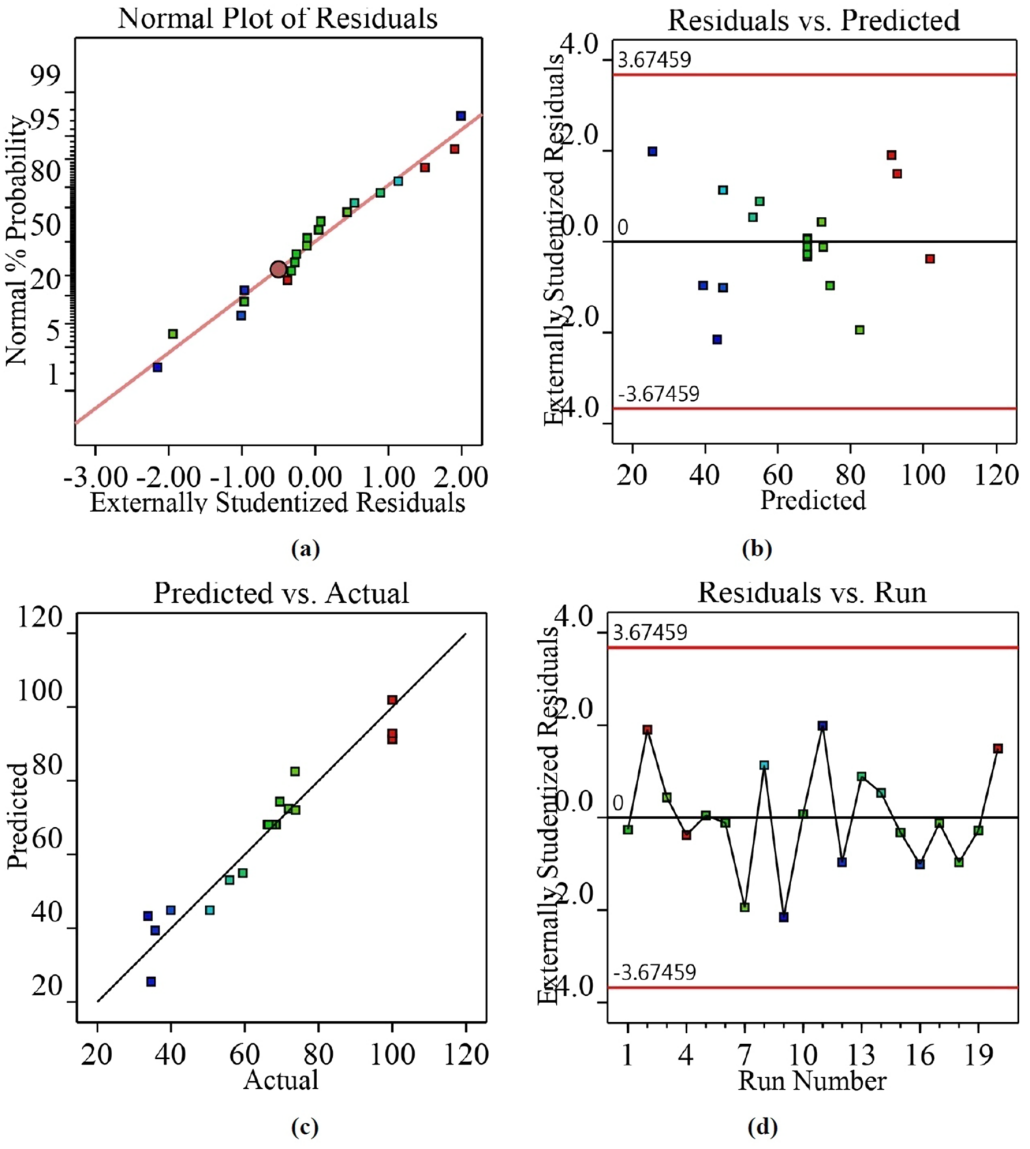
(**a**) Normal probability vs. studentized residual plot, (**b**) Studentized residuals vs. predicted. response plot, (**c**) Actual value vs. predicted value plots, (**d**) Outlier plot.

The correctness of the assumptions of ANOVA were checked by residuals that were the variations among predicted and actual values. The acceptability of the model was checked by the distribution of the residuals and was expected to follow a normal distribution if the errors were random. Studentized residuals were found out by dividing residuals by an estimate of their standard deviation. All the residuals were initially normalized with reference to their standard deviations. Studentized residuals were then fitted to the normal distribution function (Fig. 9a) and represented the normal distribution of studentized residuals. Figure 9b represents the relation between the predicted conversion versus studentized residuals. The scattered random distribution of all the residuals in Fig. 9b describes the suitability of the model to represent the process. Figure 9c indicates the actual and predicted conversions generated by the model, Eq. (4). The outlier plot in Fig. 9d represents the deviations of actual values from the predicted values. A threshold value of 3.67 standard deviations was chosen as a definition of an outlier and the majority of the standard residuals should lie between the interval of ± 3.67. Each value outside this range produced a possible error in the model. It was observed from Fig. 9d, that no data point was outside the threshold value of interval 3.67, which proved that the model was consistent with all the data.

### Optimization of reaction conditions and model verification

From the above discussion, it was possible to obtain a high conversion by searching for the optimum points. Figure 4 shows that for some combination of reaction parameters 100% conversion was possible. It could also be observed that the maximum conversion was attained either at high methanol to oil molar ratio (9) or at high reaction temperature (76 °C) or high catalyst amount (0.09). From an economic perspective, a low methanol volume was desired. Hence, optimization of the model given by Eq. (4) was performed for getting conversion close to 100%, with a minimum methanol amount, and reaction temperature of not more than 76 °C. The optimal values of the variables and their criteria, where the maximum conversion value was obtained can be seen in Table 8 (a). Similarly, to lower down the temperature further, while not exceeding the molar ratio of methanol to oil beyond 9, the optimum values and their criteria are tabulated in Table 8(b). Further, to lower down the molar ratio of catalyst to oil, without exceeding the reaction temperature and molar ratio of methanol to oil beyond 76 °C and 9 respectively, 0.078 of catalyst to oil molar ratio was obtained as optimum value for 96% conversion (Table 8c). Moreover, the model was also validated by performing the additional experiments using the optimum values of Table 8 (a–c), and the predicted model was considered to be reliable and robust.

**Table 8:** (**a**) Optimum conditions (to maximize conversion and for low methanol to oil molar ratio), related criteria, and verification of the model. (**b**) Optimum conditions (to maximize conversion and for low reaction temperature), related criteria, and verification of the model. (**c**) Optimum conditions (to maximize conversion and for a low catalyst to oil molar ratio), related criteria, and verification of the model.

Name	A: Reaction temperature (°C)	B: Molar ratio of catalyst to oil	C: Molar ratio of methanol to oil	Conversion at 60 min
(a)				
Goal	Is in range	Is in range	Is in range	Maximize
Lower limit	60	0.009	1	95
Upper limit	76	0.11	5.5	100
Lower weight	1	1	1	1
Upper weight	1	1	1	1
Importance	3	3	3	3
Optimum value by model	**76.0**	**0.110**	**4.09**	**98.48**
Experimental value (model verification)	**76.0**	**0.110**	**4.0**	**97.38**
(b)				
Goal	Is in range	Is in range	Is in range	Maximize
Lower limit	60	0.009	1	95
Upper limit	76	0.11	9	100
Lower weight	1	1	1	1
Upper weight	1	1	1	1
Importance	3	3	3	3
Optimum value by model	**69.5**	**0.110**	**8.77**	**96.69**
Experimental value (model verification)	**69**	**0.110**	**8.8**	**97.43**
(c)				
Goal	Is in range	Is in range	Is in range	Maximize
Lower limit	60	0.009	1	95
Upper limit	76	0.08	9	100
Lower weight	1	1	1	1
Upper weight	1	1	1	1
Importance	3	3	3	3
Optimum value by model	**76.0**	**0.078**	**9.0**	**96.02**
Experimental value (model verification)	**76.0**	**0.078**	**9.0**	**94.68**

Bold signify the optimum values from the model and experimentally validated values.

### The superiority of the microwave method over the conventional heating method

To determine the effect of microwave heating on the transesterification of pure triglycerides reaction and to compare it with the conventional heating method, reactions of triglycerides and methanol, using acid organocatalyst (DBSA) was carried out under microwave heating. The results obtained by using the conventional heating technique for the same factors and operating conditions have been discussed in detail in the literature^16^. With the conventional heating method, a higher conversion could be achieved in about 6 h of reaction time. Higher conversions for shorter reaction times could only be achieved at either high reaction temperature (approx. 90 °C), or higher alcohol and catalyst quantity (6:1 to 9:1 methanol-to-oil molar ratio and 0.09:1 to 0.11:1 catalyst-to-oil molar ratio). On the other hand, the microwave heating improved the reaction rate for the conversion of triglycerides into biodiesel, in the presence of DBSA catalyst and shifted the equilibrium in the direction of biodiesel production. Almost 100% biodiesel yield was achieved using less severe operating conditions, by employing microwave irradiation for only 30 min. of reaction time (Table 2, run order 4), compared to 6 h with the conventional heating technique. Microwave irradiations also enhanced biodiesel separation from the reaction mixture. The biodiesel layer separation time was reduced appreciably compared to the conventional technique. The discussed results specify that the microwave technique has a considerably lesser time of reaction and more conversion as compared to the conventional heating method. The extremely efficient reaction under microwave was due to the more efficient adsorption of the radiation directly by the OH group, which increased the temperature near the group much greater than that of its surroundings, which was far beyond the activation energy of the reaction^22^. The polarity of methanol molecules reoriented themselves, which could remove the two-tier configuration of the interface of methanol and triglycerides and make it an excellent absorbent for microwaves^18,46^. The described outcomes may be attributed to microwave localized temperature and pressure, and the microwave absorbing property to produce a volumetrically distributed heat source ^22,47–49^. Microwave heating hence outperformed the conventional heating method, providing a fast and easy way for biodiesel production.

### Properties and characterization of produced biodiesel

Table 9 shows the properties of the produced biodiesel sample (run order 4 for 30 min. in Table 2) by the pure triglycerides in the presence of 4-DBSA catalyst using methanol under microwave heating. All of the properties were determined according to the ASTM standards. Although some of the properties were higher in comparison to petrodiesel but within the ASTM standards. The flashpoint of produced biodiesel was high which is helpful for safe transportation. The cetane number was also higher than petrodiesel. All the tested properties of produced biodiesel were within the ASTM standards.

**Table 9:** Properties of produced biodiesel with test methods and limits as per ASTM D6751.

Property	Test method used	Limits for FAME (B100) as per ASTM D6751	Specifications of produced biodiesel
Flashpoint (°C)	ASTM D93	> 130	161
Kinematic viscosity at 40 °C, (mm^2^/s)	ASTM D445	1.9–6.0	4.55
Specific gravity at 15 °C (g/cc)	ASTM D4052	0.87–0.90	0.879
Carbon residue (% m/m)	ASTM D4530	0.05 max	0.021
Cloud point (°C)	ASTM D97	− 3 to 12	2
Cetane number	ASTM D613	47 min	51
Acid value (mg/KOH)	ASTM D974	0.5 max	0.05
Distillation, 90% recovery (°C)	ASTM D1160	360 max	345
Copper strip corrosion 3 h at 50 °C	ASTM D130	1 max	1b

## Conclusions

To enhance the rate of the transesterification reaction, the biodiesel was produced by the pure triglycerides in the presence of a 4-DBSA catalyst using methanol under microwave heating. To make the biodiesel production process cost-efficient, optimization of parameters was performed using Design Expert 11 software, and a set of 20 experiments were designed from the RSM technique to determine the effect of reaction temperature, the molar ratio of catalyst to oil, and the molar ratio of methanol to oil on the conversion of triglycerides. The rate of transesterification reaction for biodiesel production using pure triglycerides in the presence of DBSA catalyst using microwave heating was much higher than with the same catalyst under the conventional heating method. By using this catalyst under microwave heating at a temperature of 76 °C, conversion close to 100% in only 30 min of reaction time was obtained using a 0.09 molar ratio of catalyst to oil and 9.0 molar ratio of methanol to oil (Table 2, run order 4). Whereas, when DBSA was used as a catalyst under the conventional heating method, the same conversion (close to 100%) under similar operating conditions was achieved in 6 h of reaction time^16^. The conversion close to 100% could also be achieved in the present work with very less amount of methanol (4.09 mol%), a slightly higher catalyst amount (0.11 mol%), and in 60 min of reaction time (Table 8a). The drastic reduction in reaction time from 6 h to 30 min indicated the superiority of microwave heating over conventional heating method for the transesterification of pure triglycerides in the presence of DBSA catalyst. The slow rate of reaction which was the main hurdle for the commercialization of the DBSA catalyst for the production of biodiesel from low-cost high FFA oil, has now been combated by using microwave heating. As the DBSA catalyst is less corrosive its use also reduces the cost of equipment. In this study, a modified polynomial model was developed with an F-value of 51.49 (*p* value < 0.0001) and R^2^ value of 0.9321 (Table 10), which implied that the selected model was adequately fitted with the experimental data and could be used for understanding the effect of various process parameters. From the variables studied, the catalyst to oil molar ratio and reaction temperature created a stronger effect on the biodiesel production than those exhibited by the methanol to oil molar ratio. By the validation of experiments, the adequacy/fit of the model employed was justified, suggesting its relevance for a reliable prediction of biodiesel production from triglycerides and methanol using acid organocatalyst (4-dodecyl benzene sulfonic acid) under microwave heating. Evaluation of the experimental and predicted values showed good agreement between them, and it could effectively describe the relationship between the factors and response. The DBSA being a less corrosive and strong catalyst performed both esterification and transesterification simultaneously in biodiesel production from low-cost non-edible oil with a faster rate of a reaction under mild operating conditions. The microwave was an energy-efficient method for the transesterification process, as it enhanced the transesterification process, lowered down the cost of processing, had no need for higher temperature, and needed less severe operating conditions. Thus, it was much better than the conventional heating method. Also, the produced biodiesel was of good quality, as all the properties were within the prescribed limits of the ASTM D 6751 standard.

**Table 10:** ANOVA for the modified quadratic polynomial model of 60 min reaction time.

Source	Sum of squares	DF	Mean square	F-value	*P*-value	
**Model**	7122.51	4	1780.63	51.49	< 0.0001	Significant
A-Reaction temperature	2596.69	1	2596.69	75.09	< 0.0001	Significant
B-Catalyst to oil molar ratio	2962.37	1	2962.37	85.67	< 0.0001	Significant
C-Methanol to oil molar ratio	1283.38	1	1283.38	37.11	< 0.0001	Significant
C^2^	280.06	1	280.06	8.10	0.0123	Significant
**Residual**	518.71	15	34.58			
Lack of fit	513.63	10	51.36	50.60	0.0002	Significant
Pure error	5.08	5	1.02			
**Cor total**	7641.21	19				

R^2^ = 0.9321.

Adjusted R^2^ = 0.9140.

Predicted R^2^ = 0.8613.

In the production of biodiesel from triglycerides and methanol using acid organocatalyst, future research work is quite important to determine the effect of other new technologies such as ultrasonic, microchannel reactor, and static mixers. Besides that, the kinetics of transesterification between triglycerides and acid organocatalyst under microwave irradiations have not been studied until now. This will help widen the work and make an improved prediction for any change in the system.

## Supplementary information

Supplementary Information.
