# Supplementary material for: Intensification and optimization of biodiesel production using microwave-assisted acid-organo catalyzed transesterification process
Source: Sci Rep. 2020 Dec 4;10:21239. doi: 10.1038/s41598-020-77798-1 (PMC7718927; doi:10.1038/s41598-020-77798-1)
Supplement: Supplementary file 1 — Supplementary Information. [file 41598_2020_77798_MOESM1_ESM.pdf]

Supplementary files

Intensification and optimization of biodiesel production using microwave-assisted acid-organo catalyzed transesterification process

Moina Athar[1], Sadaf Zaidi[2*], Saeikh Zaffar Hassan[1]
[1]*Department of Petroleum Studies, Zakir Husain College of Engineering and Technology, Aligarh Muslim University, Aligarh, India*
[2]*Department of Post Harvest Engineering and Technology, Faculty of Agricultural Sciences, Aligarh Muslim University, Aligarh, India*
***E mail:* sadaf63in@yahoo.com**

Table S1. ANOVA for the quadratic polynomial model of 10 minutes reaction time.

Source	Sum of Squares	df	Mean Square	F-value	p-value	
**Model**	3839.58	9	426.62	13.17	0.0002	significant
A-Reaction Temperature	828.03	1	828.03	25.56	0.0005	
B-Catalyst to oil molar ratio	1502.77	1	1502.77	46.40	< 0.0001	
C-Methanol to oil molar ratio	938.75	1	938.75	28.98	0.0003	
AB	21.37	1	21.37	0.6597	0.4356	
AC	28.67	1	28.67	0.8851	0.3690	
BC	295.80	1	295.80	9.13	0.0129	
A^2	216.25	1	216.25	6.68	0.0272	
B^2	14.19	1	14.19	0.4381	0.5230	
C^2	10.59	1	10.59	0.3269	0.5801	
**Residual**	323.90	10	32.39			
Lack of Fit	322.10	5	64.42	178.75	< 0.0001	significant
Pure Error	1.80	5	0.3604			
**Cor Total**	4163.48	19				

R^2 =0.9222
Adjusted R^2=0.8522
Predicted R^2=0.4042

Table S2. ANOVA for the quadratic polynomial model of 30 minutes reaction time.

Source	Sum of Squares	df	Mean Square	F-value	p-value	
**Model**	7366.43	9	818.49	14.50	0.0001	significant
A-Reaction Temperature	1945.11	1	1945.11	34.46	0.0002	
B-Catalyst to oil molar ratio	2904.64	1	2904.64	51.46	< 0.0001	
C-Methanol to oil molar ratio	2068.68	1	2068.68	36.65	0.0001	
AB	0.7357	1	0.7357	0.0130	0.9114	
AC	209.16	1	209.16	3.71	0.0831	
BC	74.92	1	74.92	1.33	0.2761	
A^2	110.78	1	110.78	1.96	0.1915	
B^2	26.12	1	26.12	0.4627	0.5118	
C^2	10.48	1	10.48	0.1856	0.6757	
**Residual**	564.47	10	56.45			
Lack of Fit	560.39	5	112.08	137.39	< 0.0001	significant
Pure Error	4.08	5	0.8158			
**Cor Total**	7930.90	19				

R^2 =0.9288
Adjusted R^2=0.8648
Predicted R^2=0.4503

Table S3. ANOVA for the quadratic polynomial model of 120 minutes reaction time.

Source	Sum of Squares	df	Mean Square	F-value	p-value	
**Model**	5499.74	9	611.08	6.25	0.0042	significant
A-Reaction Temperature	1633.67	1	1633.67	16.71	0.0022	
B-Catalyst to oil molar ratio	1954.45	1	1954.45	19.99	0.0012	
C-Methanol to oil molar ratio	1476.38	1	1476.38	15.10	0.0030	
AB	193.20	1	193.20	1.98	0.1901	
AC	1.80	1	1.80	0.0184	0.8949	
BC	45.44	1	45.44	0.4648	0.5109	
A^2	185.60	1	185.60	1.90	0.1983	
B^2	5.52	1	5.52	0.0564	0.8170	
C^2	1.95	1	1.95	0.0200	0.8904	
**Residual**	977.64	10	97.76			
Lack of Fit	972.01	5	194.40	172.67	< 0.0001	significant
Pure Error	5.63	5	1.13			
**Cor Total**	6477.39	19				

R^2 =0.8491
Adjusted R^2=0.7132
Predicted R^2=-0.1466

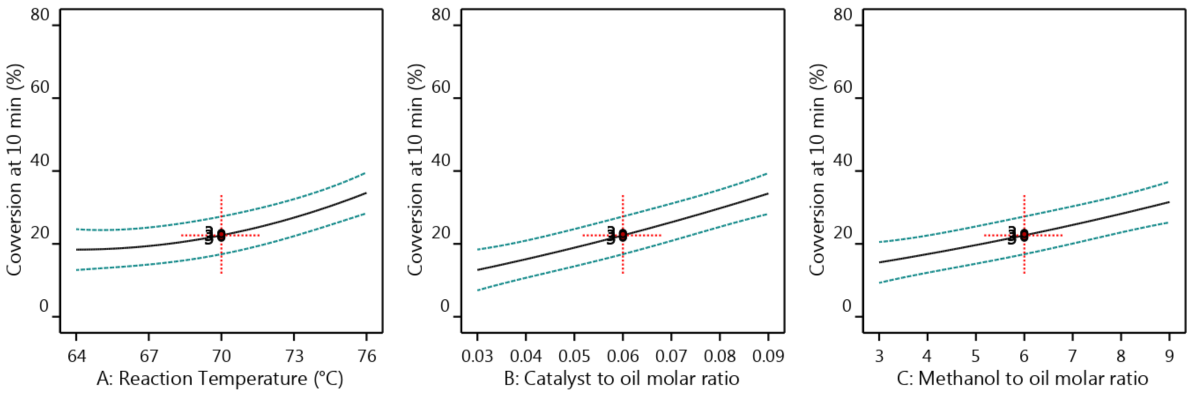

(a) (b) (c)

Figure S1. Main effects of factors on the conversion of triglyceride to FAME at 10 minutes of reaction time (a) Effect of reaction temperature(A) [At B=0.06 and C=6] (b)Effect of Catalyst to oil molar ratio(B)[At A=70°C and C=6] (c)Effect of methanol to oil molar ratio(C)[A=70°C and B=0.06].

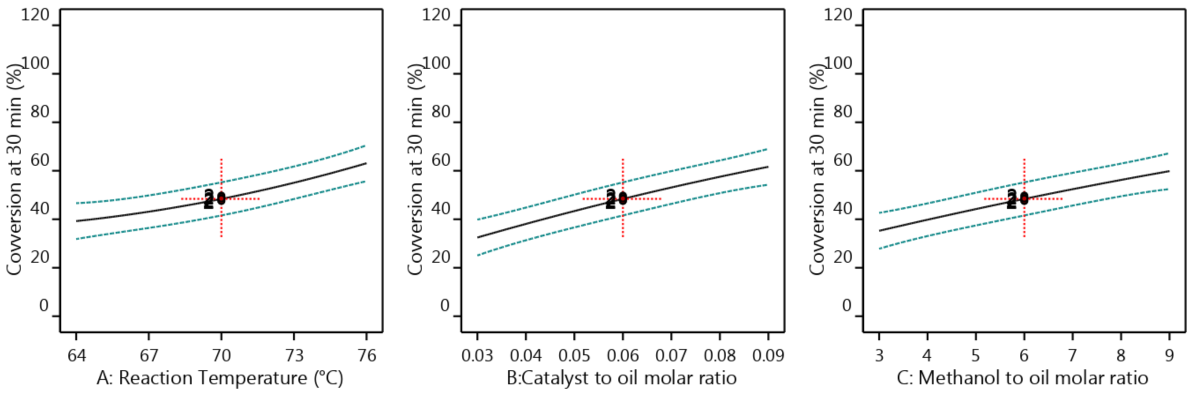

(a) (b) (c)

Figure S2. Main effects of factors on the conversion of triglyceride to FAME at 30 minutes of reaction time (a) Effect of reaction temperature(A) [At B=0.06 and C=6] (b)Effect of Catalyst to oil molar ratio(B)[At A=70°C and C=6] (c)Effect of methanol to oil molar ratio(C)[A=70°C and B=0.06].

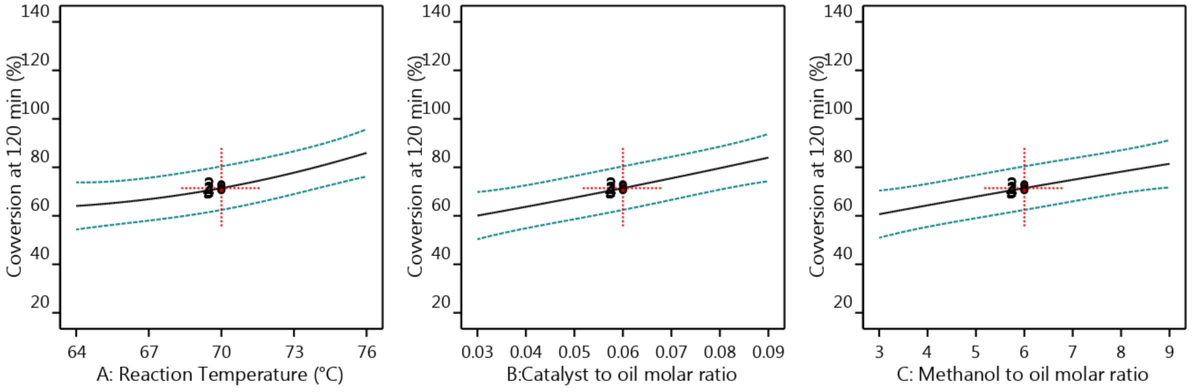

(a) **(b)** **(c)**

Figure S3. Main effects of factors on the conversion of triglyceride to FAME at 120 minutes of reaction time (a) Effect of reaction temperature(A) [At B=0.06 and C=6] (b)Effect of Catalyst to oil molar ratio(B)[At A=70°C and C=6] (c)Effect of methanol to oil molar ratio(C)[A=70°C and B=0.06].

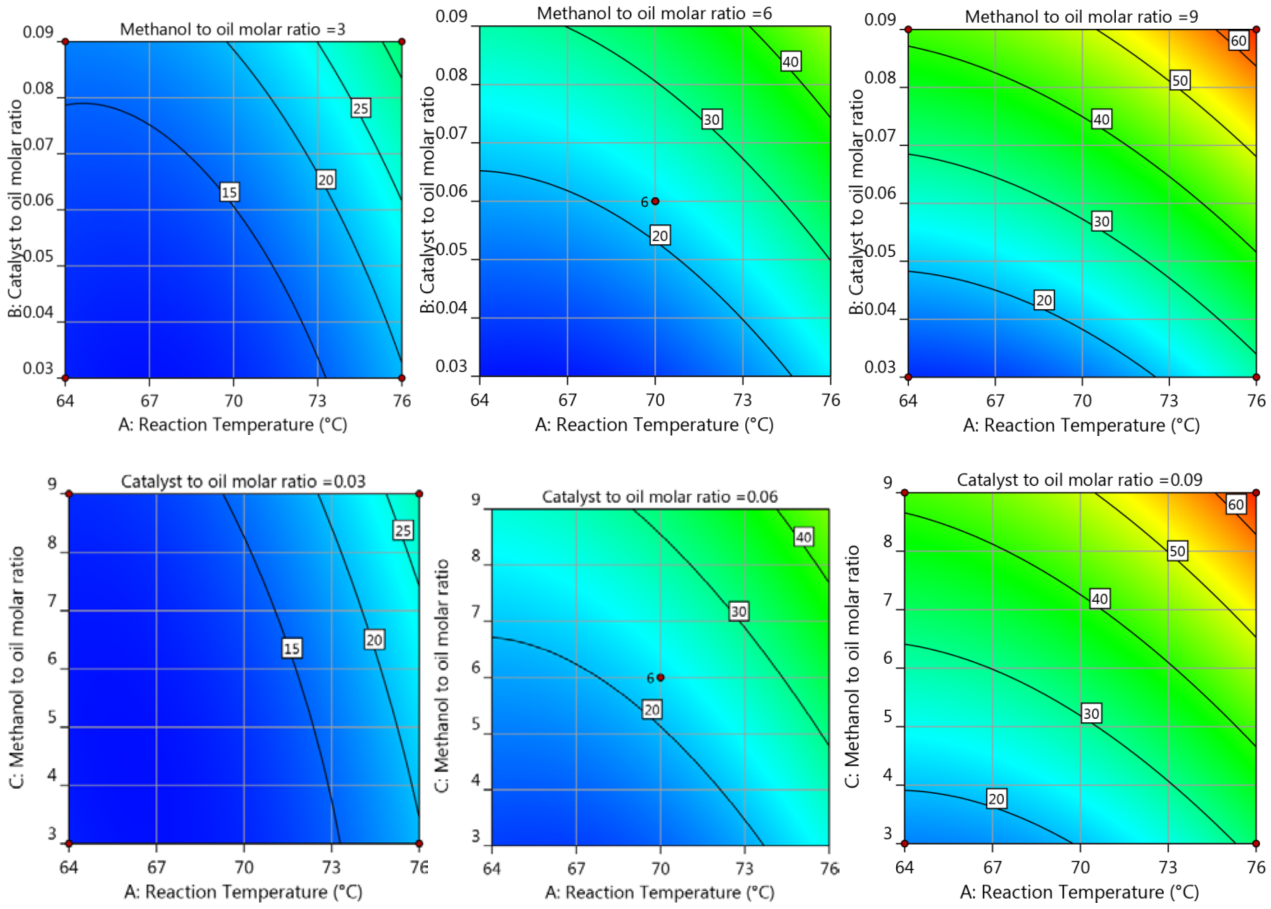

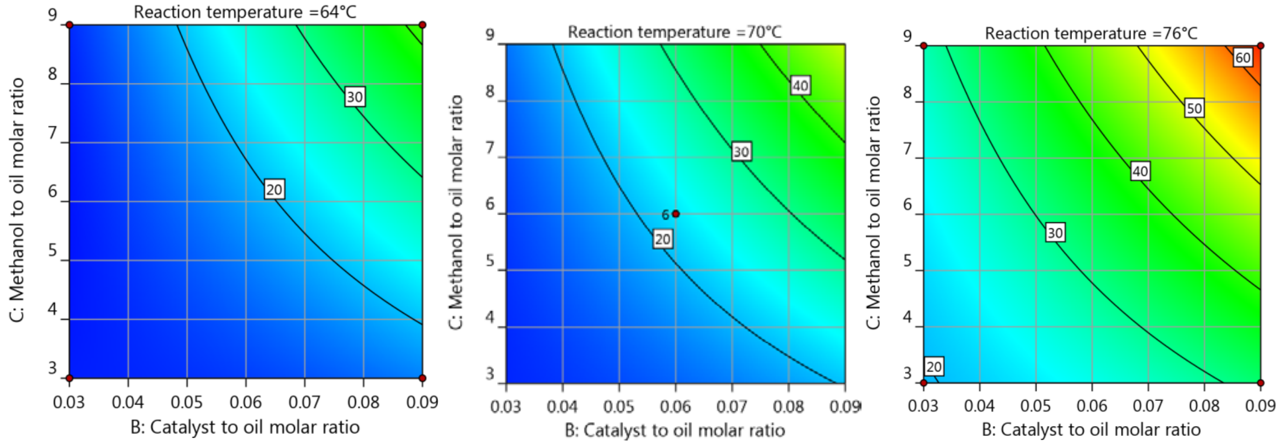

Figure S4. Contour plots for 10 min. of reaction at lower(-1),middle(0) and upper limits(+1) of fixed factors (a)Reaction temperature-Catalyst to oil molar ratio (b) Reaction temperature-Methanol to oil molar ratio (c) Catalyst to oil molar ratio- Methanol to oil molar ratio.

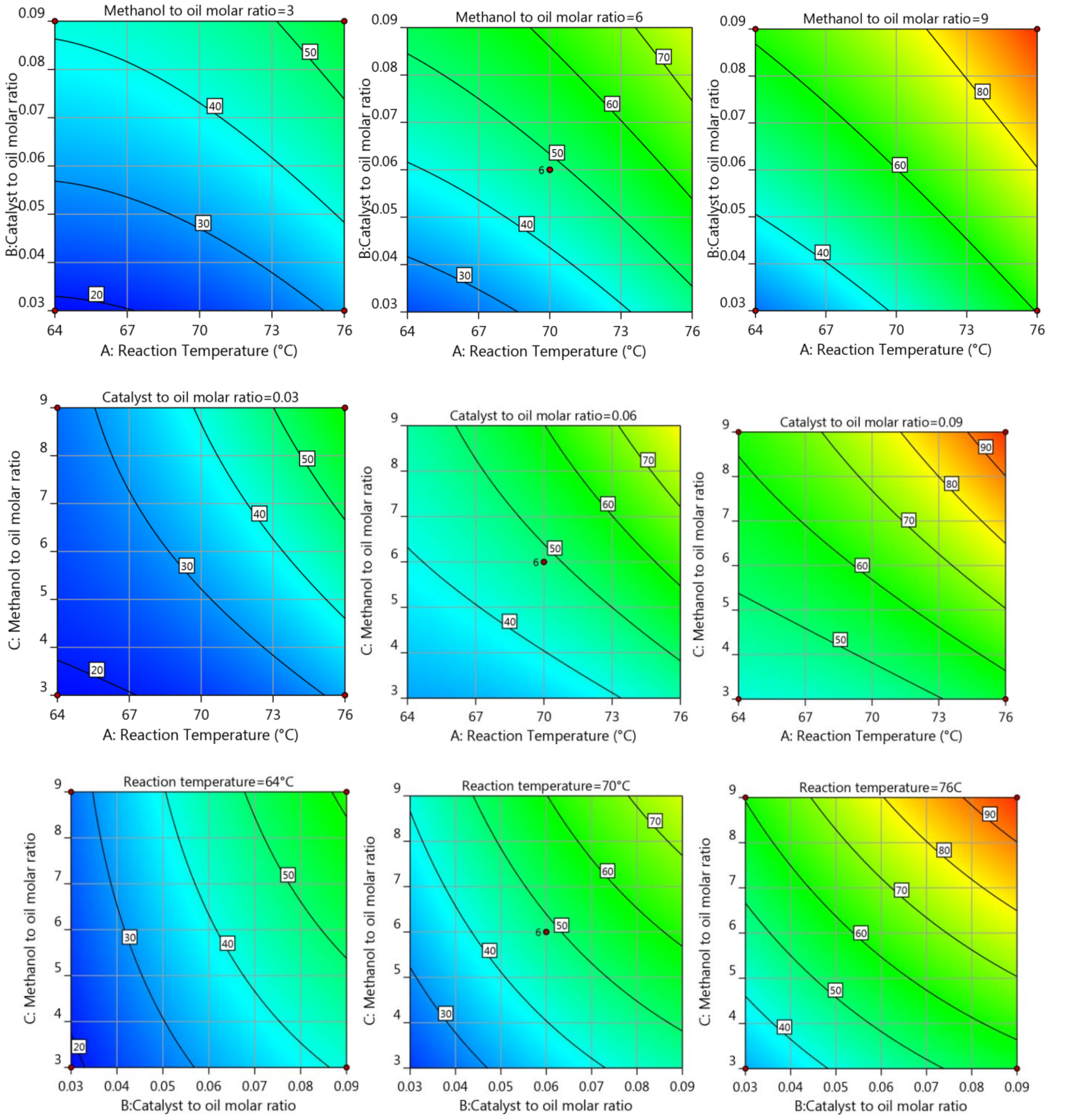

Figure S5. Contour plots for 30 min. of reaction at lower(-1),middle(0) and upper limits(+1) of fixed factors (a)Reaction temperature-Catalyst to oil molar ratio (b) Reaction temperature-Methanol to oil molar ratio (c) Catalyst to oil molar ratio- Methanol to oil molar ratio**.**

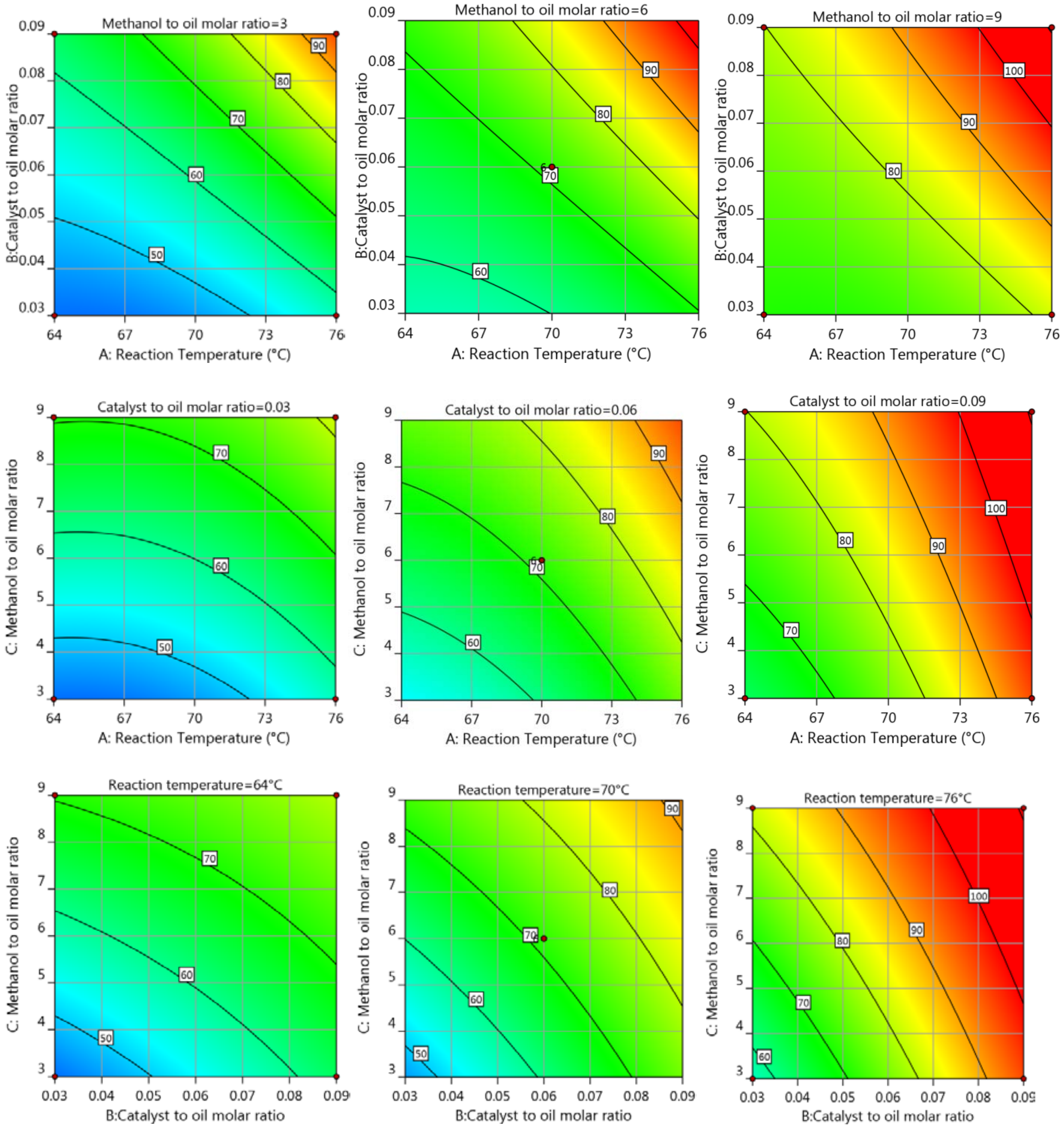

Figure S6. Contour plots for 120 min. of reaction at lower(-1),middle(0) and upper limits(+1) of fixed factors (a)Reaction temperature-Catalyst to oil molar ratio (b) Reaction temperature-Methanol to oil molar ratio (c) Catalyst to oil molar ratio- Methanol to oil molar ratio.

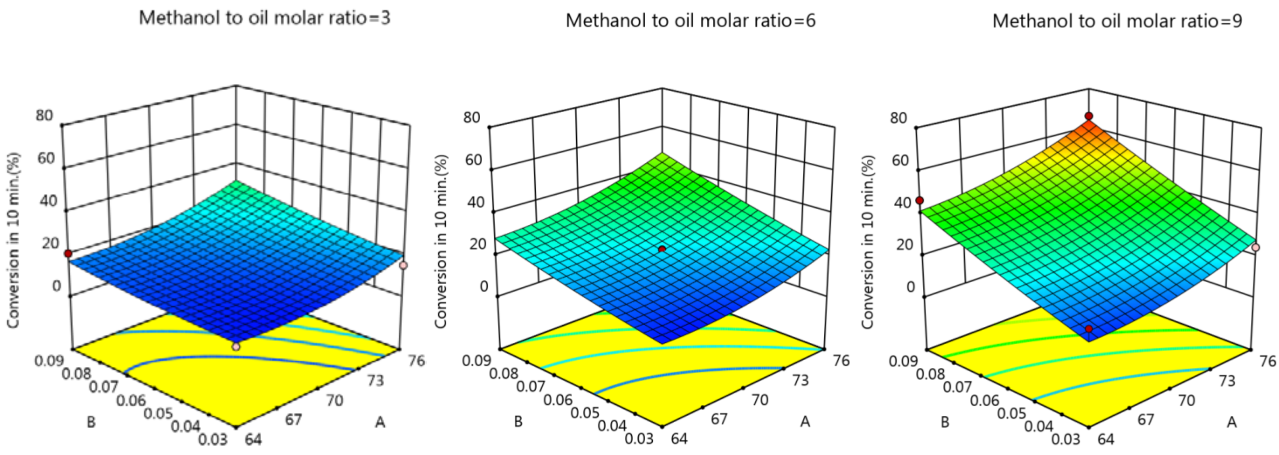

(a)

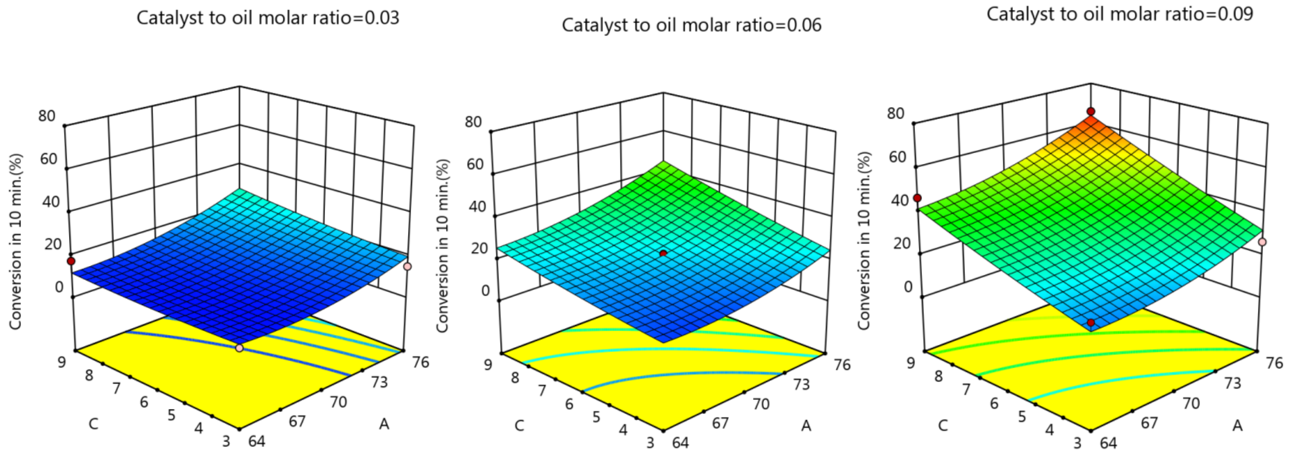

(b)

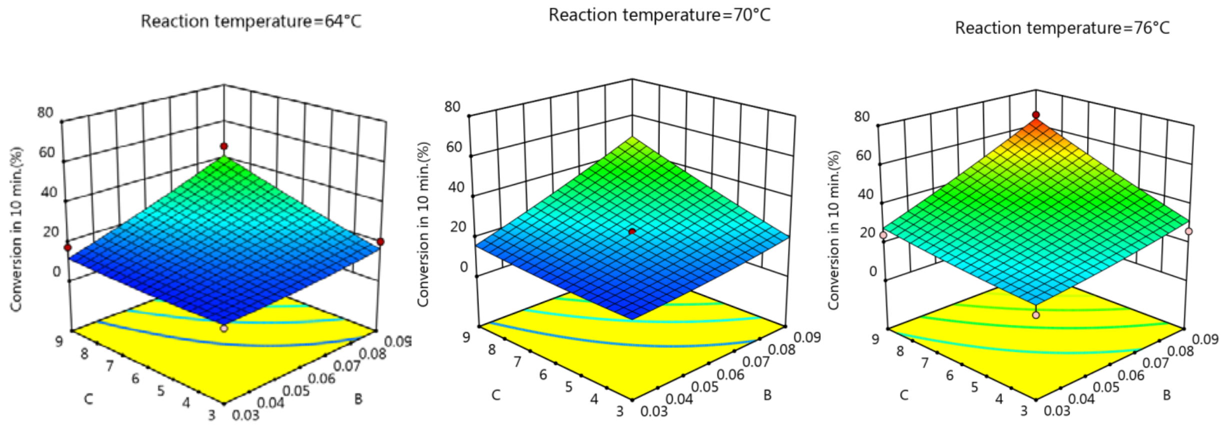

(c)

Figure S7. Response surface plots for 10 min. of reaction at lower(-1), middle(0) and upper limits(+1) of fixed factors (a) Reaction temperature(A)-Catalyst to oil molar ratio(B) (b) Reaction temperature(A)-Methanol to oil molar ratio(C) (c) Catalyst to oil molar ratio(B)- Methanol to oil molar ratio(C).

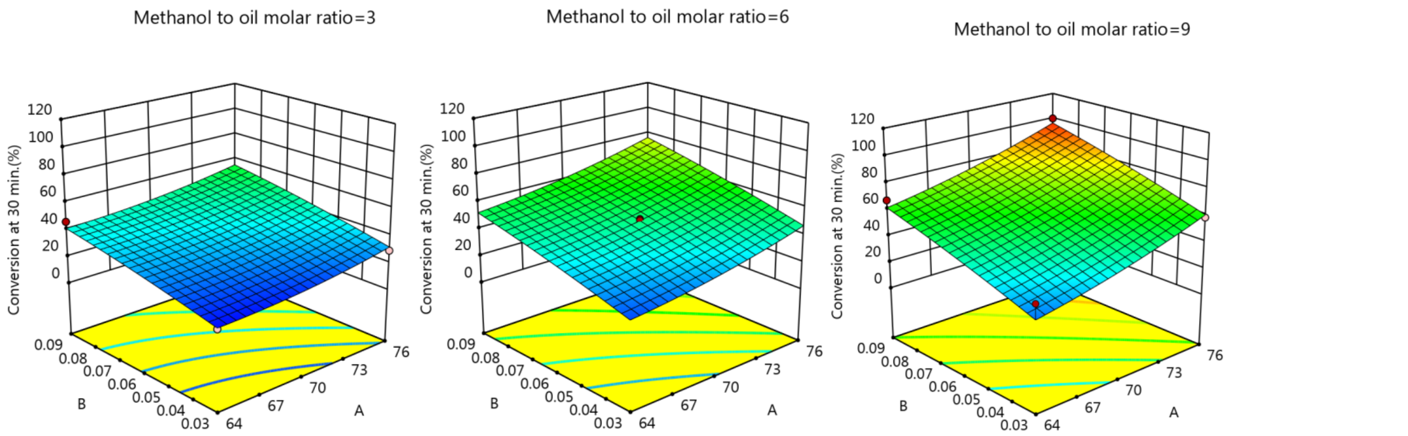

(a)

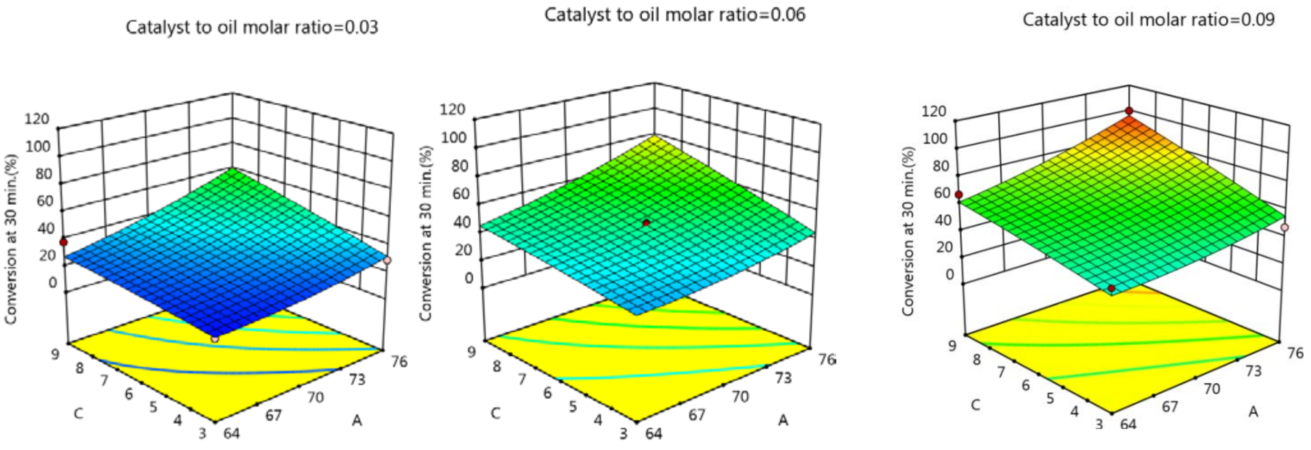

(b)

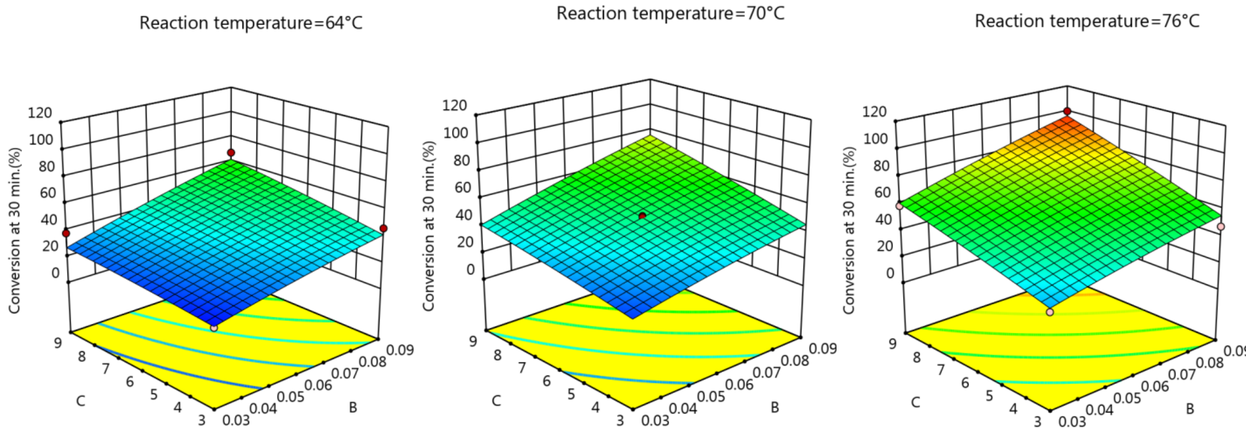

(c)

Figure S8. Response surface plots for 30 min. of reaction at lower(-1), middle(0) and upper limits(+1) of fixed factors (a) Reaction temperature(A)-Catalyst to oil molar ratio(B) (b) Reaction temperature(A)-Methanol to oil molar ratio(C) (c) Catalyst to oil molar ratio(B)- Methanol to oil molar ratio(C).

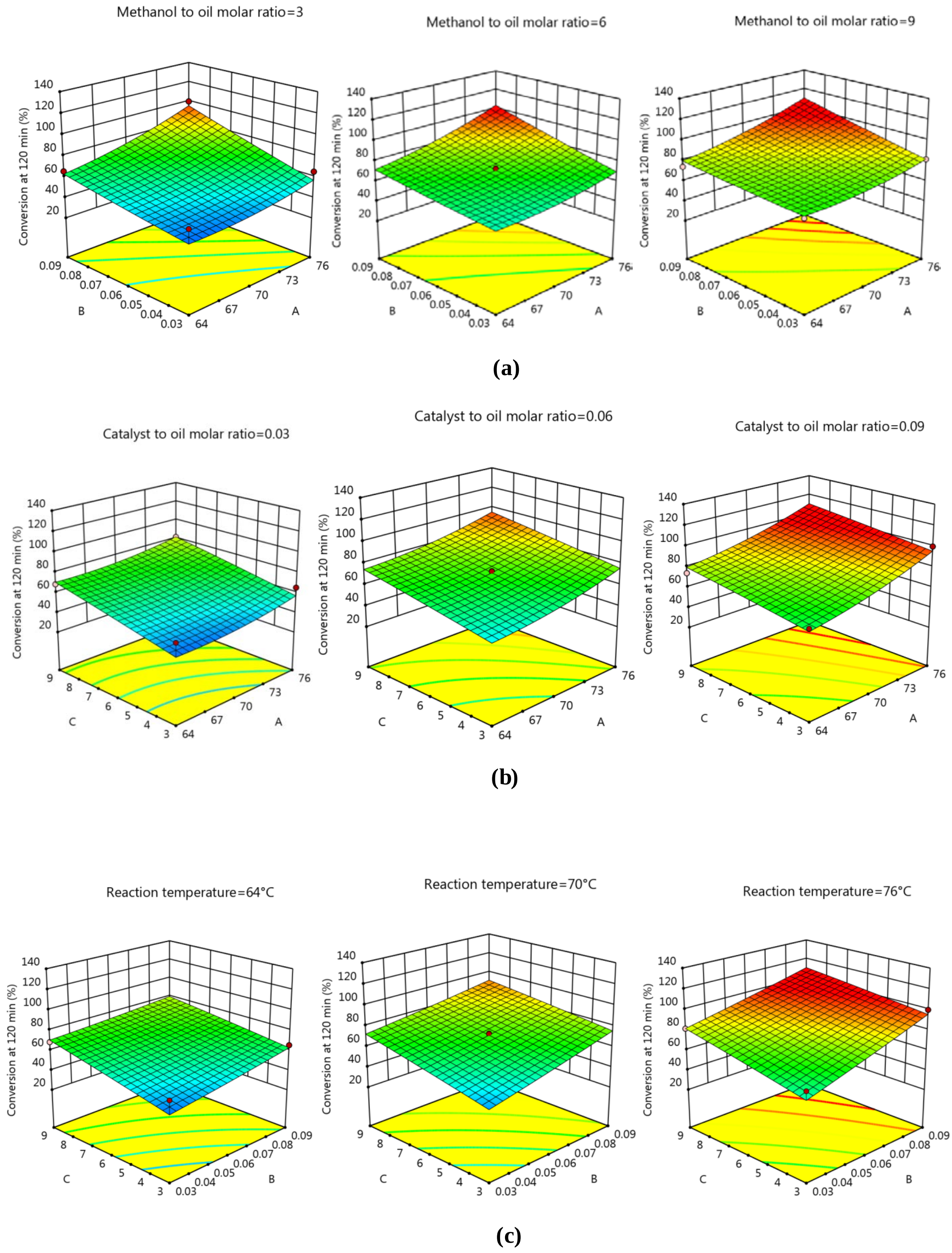

Figure S9. Response surface plots for 120 min. of reaction at lower(-1), middle(0) and upper limits(+1) of fixed factors (a) Reaction temperature(A)-Catalyst to oil molar ratio(B) (b) Reaction temperature(A)-Methanol to oil molar ratio(C) (c) Catalyst to oil molar ratio(B)- Methanol to oil molar ratio(C).

Table S4. ANOVA for the factorial model of 10 minutes reaction time.

Source	Sum of Squares	df	Mean Square	F-value	p-value	
**Model**	2484.15	6	414.02	115.77	< 0.0001	significant
A-Reaction Temperature	175.28	1	175.28	49.01	0.0004	

B-Catalyst to oil molar ratio	1093.72	1	1093.72	305.82	< 0.0001	
C-Methanol to oil molar ratio	869.32	1	869.32	243.07	< 0.0001	
AB	21.37	1	21.37	5.97	0.0502	
AC	28.67	1	28.67	8.02	0.0299	
BC	295.80	1	295.80	82.71	< 0.0001	
Curvature	116.77	1	116.77	32.65	0.0012	
**Residual**	21.46	6	3.58			
Lack of Fit	19.66	1	19.66	54.54	0.0007	significant
Pure Error	1.80	5	0.3604			
**Cor Total**	2622.37	13				

R^2 = 0.9914
Adjusted R^2= 0.9829
Predicted R^2= 0.4969

Table S5. ANOVA for the factorial model of 30 minutes reaction time.

Source	Sum of Squares	df	Mean Square	F-value	p-value	
**Model**	4447.02	6	741.17	66.82	< 0.0001	significant
A-Reaction Temperature	539.56	1	539.56	48.64	0.0004	
B-Catalyst to oil molar ratio	1719.44	1	1719.44	155.01	< 0.0001	
C-Methanol to oil molar ratio	1903.20	1	1903.20	171.58	< 0.0001	
AB	0.7357	1	0.7357	0.0663	0.8054	
AC	209.16	1	209.16	18.86	0.0049	
BC	74.92	1	74.92	6.75	0.0407	
Curvature	12.58	1	12.58	1.13	0.3279	
**Residual**	66.55	6	11.09			
Lack of Fit	62.47	1	62.47	76.58	0.0003	significant
Pure Error	4.08	5	0.8158			
**Cor Total**	2622.37	13				

R^2 = 0.9853
Adjusted R^2= 0.9705
Predicted R^2= 0.1129

Table S6. ANOVA for the factorial model of 120 minutes reaction time.

Source	Sum of Squares	df	Mean Square	F-value	p-value	
**Model**	1798.29	6	299.71	63.01	< 0.0001	significant
A-Reaction Temperature	828.45	1	828.45	174.17	< 0.0001	
B-Catalyst to oil molar ratio	563.84	1	563.84	118.54	< 0.0001	
C-Methanol to oil molar ratio	165.57	1	165.57	34.81	0.0011	
AB	193.20	1	193.20	40.62	0.0007	
AC	1.80	1	1.80	0.3775	0.5615	
BC	45.44	1	45.44	9.55	0.0214	
Curvature	85.46	1	85.46	17.97	0.0054	
**Residual**	28.54	6	4.76			
Lack of Fit	22.91	1	22.91	20.35	0.0063	significant
Pure Error	5.63	5	1.13			
**Cor Total**	1912.29	13				

R^2 = 0.9844
Adjusted R^2= 0.9688
Predicted R^2= 0.1930

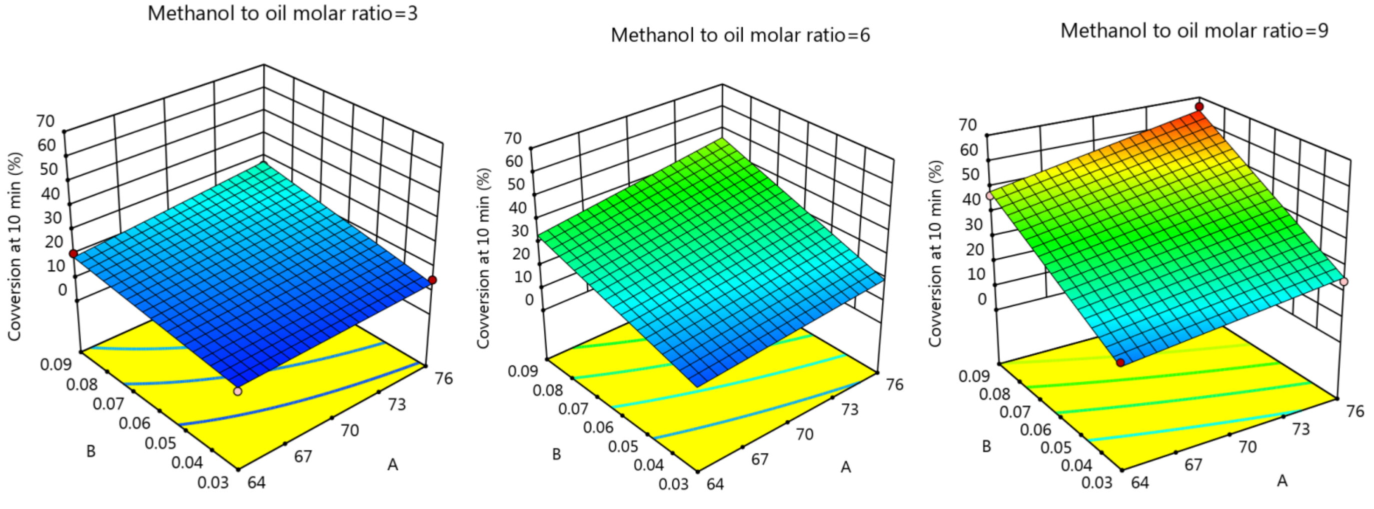

(a)

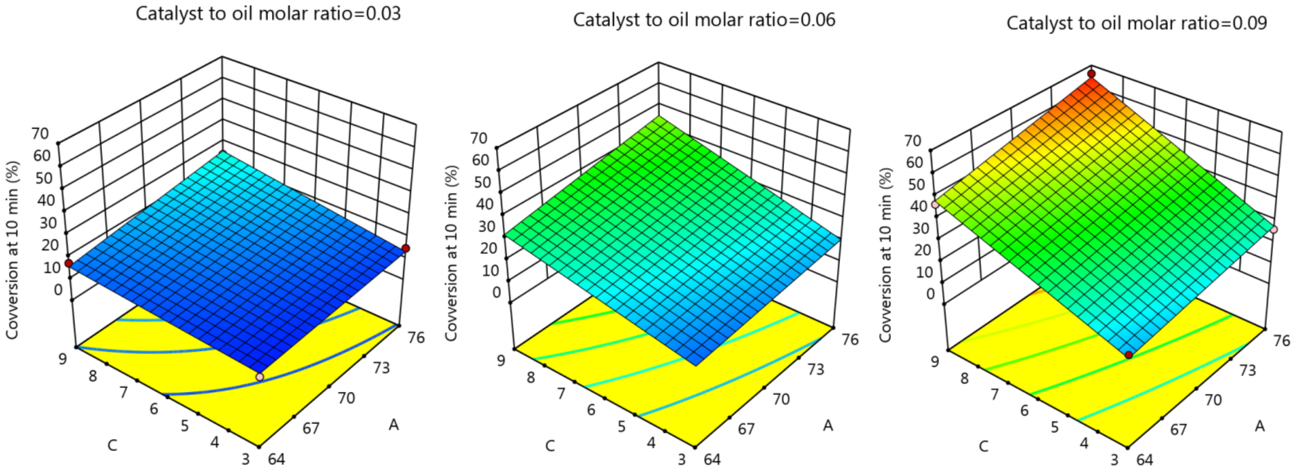

(b)

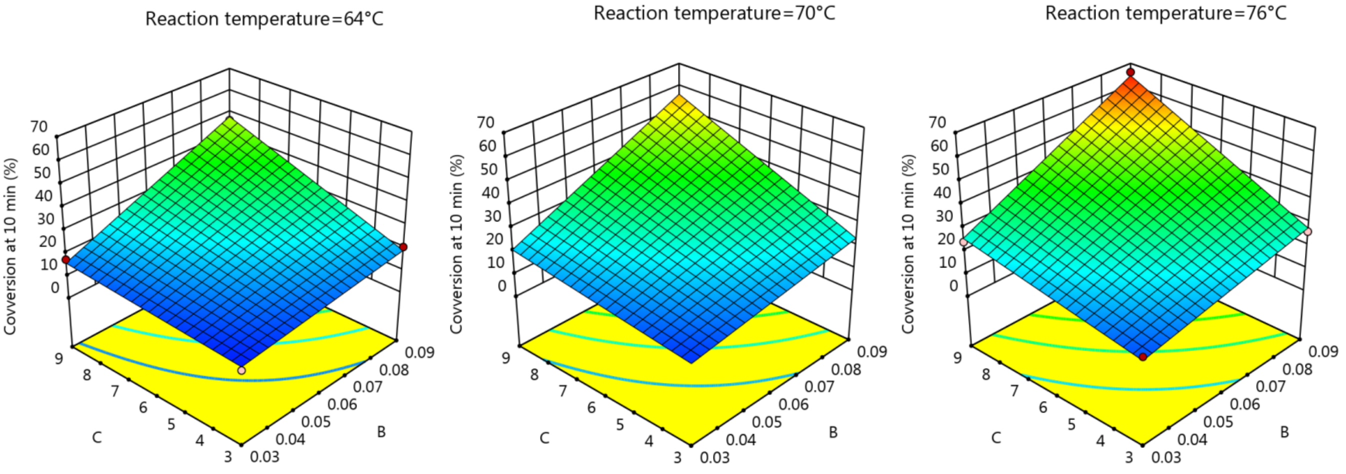

(c)

Figure S10. Surface plots factorial design for 10 min. of reaction at lower(-1), middle(0) and upper limits(+1) of fixed factors (a) Reaction temperature(A)-Catalyst to oil molar ratio(B) (b) Reaction temperature(A)-Methanol to oil molar ratio(C) (c) Catalyst to oil molar ratio(B)- Methanol to oil molar ratio(C).

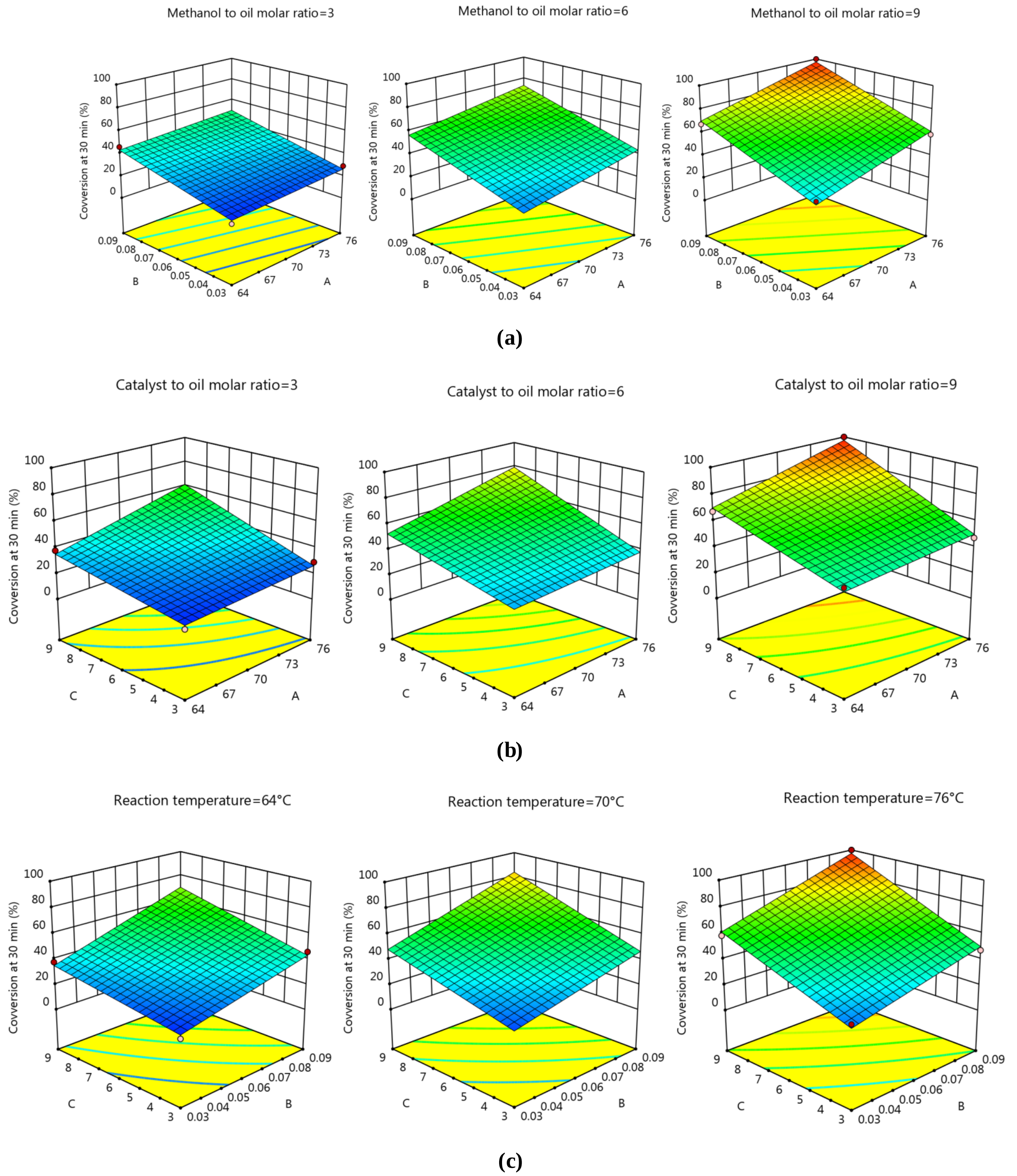

Figure S11. Surface plots factorial design for 30 min. of reaction at lower(-1), middle(0) and upper limits(+1) of fixed factors (a) Reaction temperature(A)-Catalyst to oil molar ratio(B) (b) Reaction temperature(A)-Methanol to oil molar ratio(C) (c) Catalyst to oil molar ratio(B)- Methanol to oil molar ratio(C).

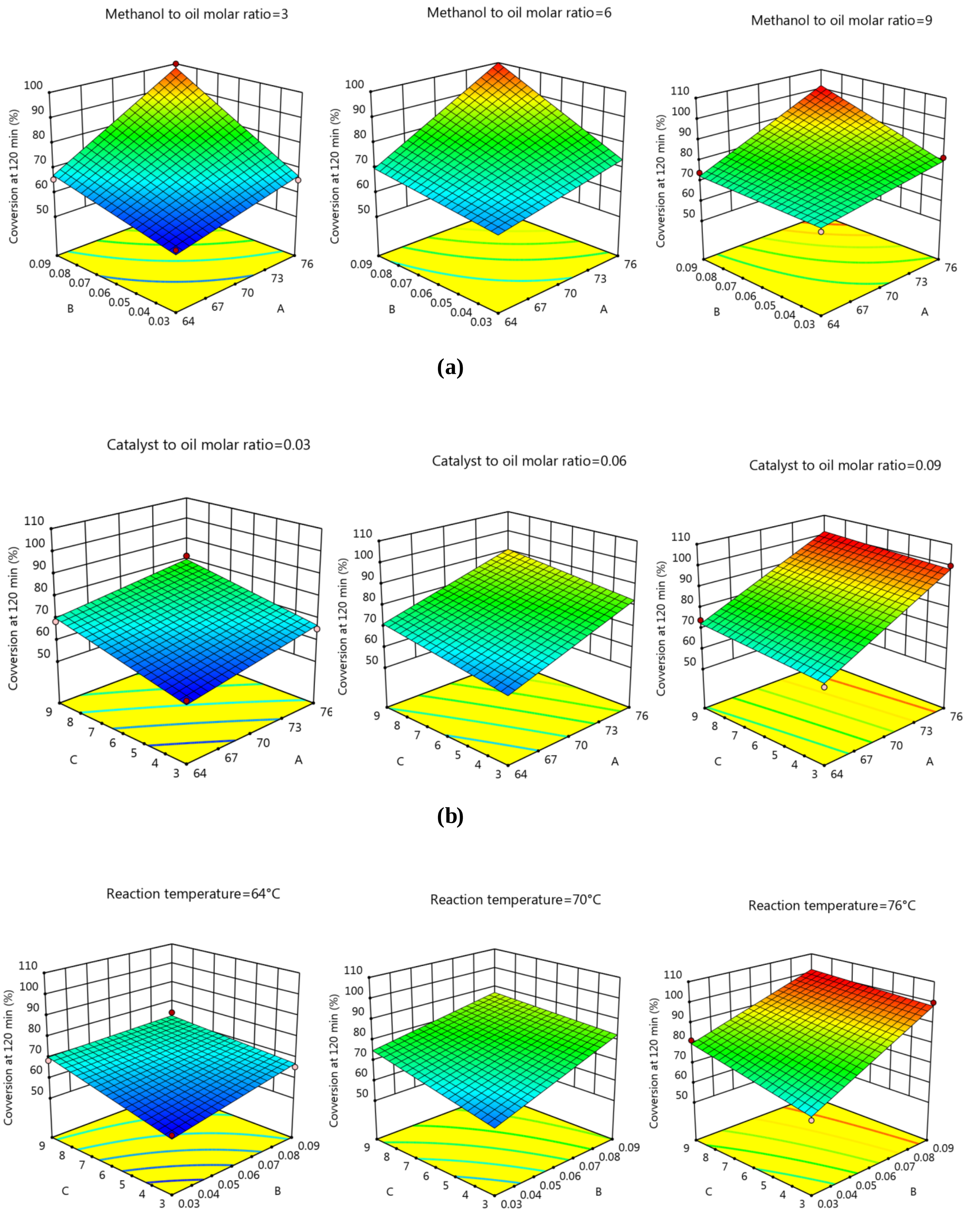

Figure S12. Surface plots factorial design for 120 min. of reaction at lower(-1), middle(0) and upper limits(+1) of fixed factors (a) Reaction temperature(A)-Catalyst to oil molar ratio(B) (b) Reaction temperature(A)-**Methanol to oil** molar ratio(C) (c) Catalyst to oil molar ratio(B)- Methanol to oil molar ratio(C).

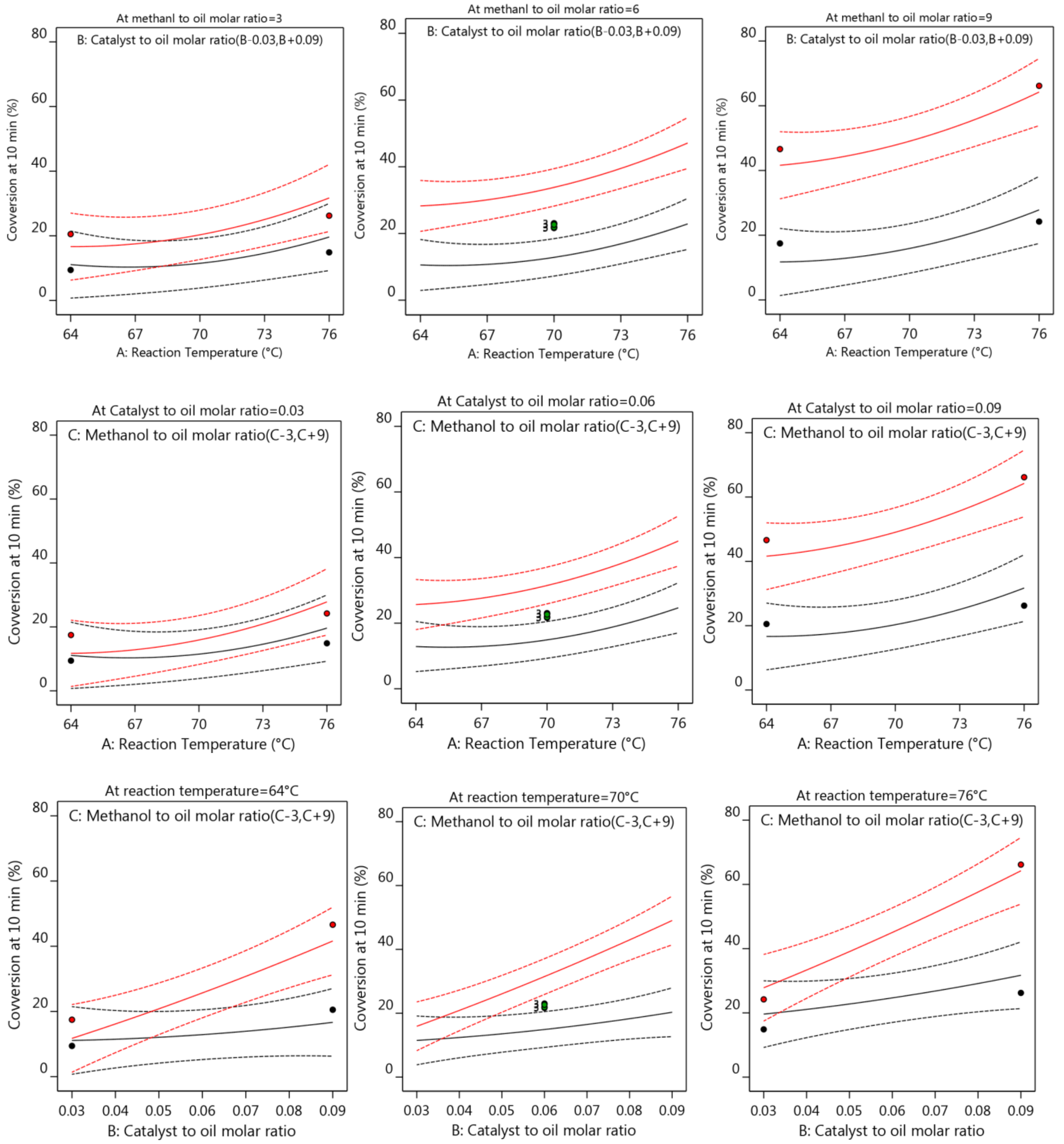

Figure S13. Interaction effects of factors on the conversion of triglyceride to FAME at 10 minutes of reaction time (a) Reaction temperature-Catalyst to oil molar ratio(B- 0.03 B+ 0.09),(b) Reaction temperature-Methanol to oil molar ratio(C- 3 ,C+ 9),(c) Catalyst to oil molar ratio- Methanol to oil molar ratio(C- 3,C+ 9).

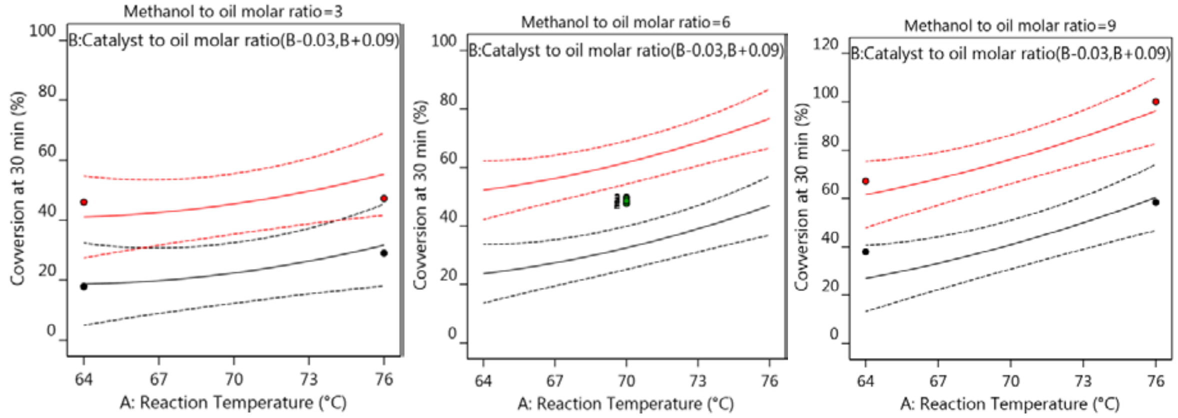

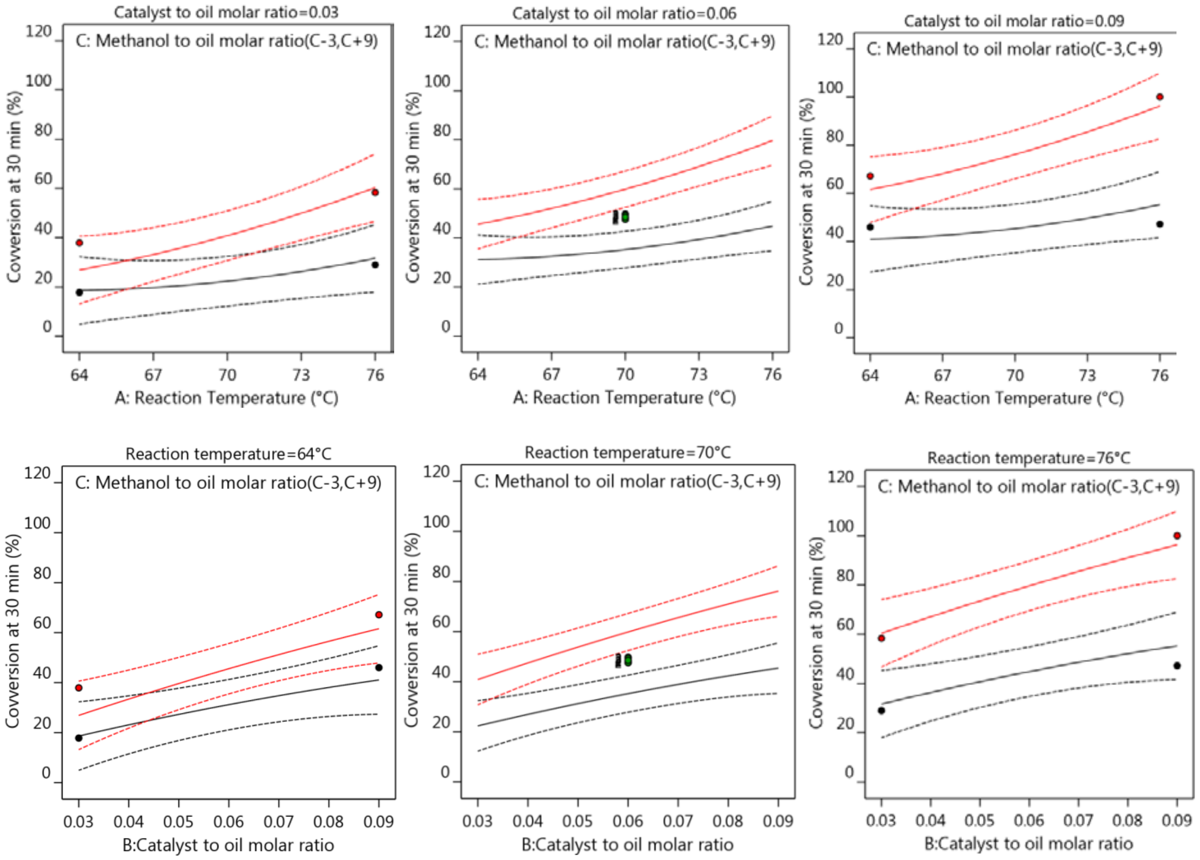

Figure S14. Interaction effects of factors on the conversion of triglyceride to FAME at 30 minutes of reaction time (a) Reaction temperature-Catalyst to oil molar ratio(B- 0.03 B+ 0.09),(b) Reaction temperature-Methanol to oil molar ratio(C- 3 ,C+ 9),(c) Catalyst to oil molar ratio- Methanol to oil molar ratio(C- 3,C+ 9).

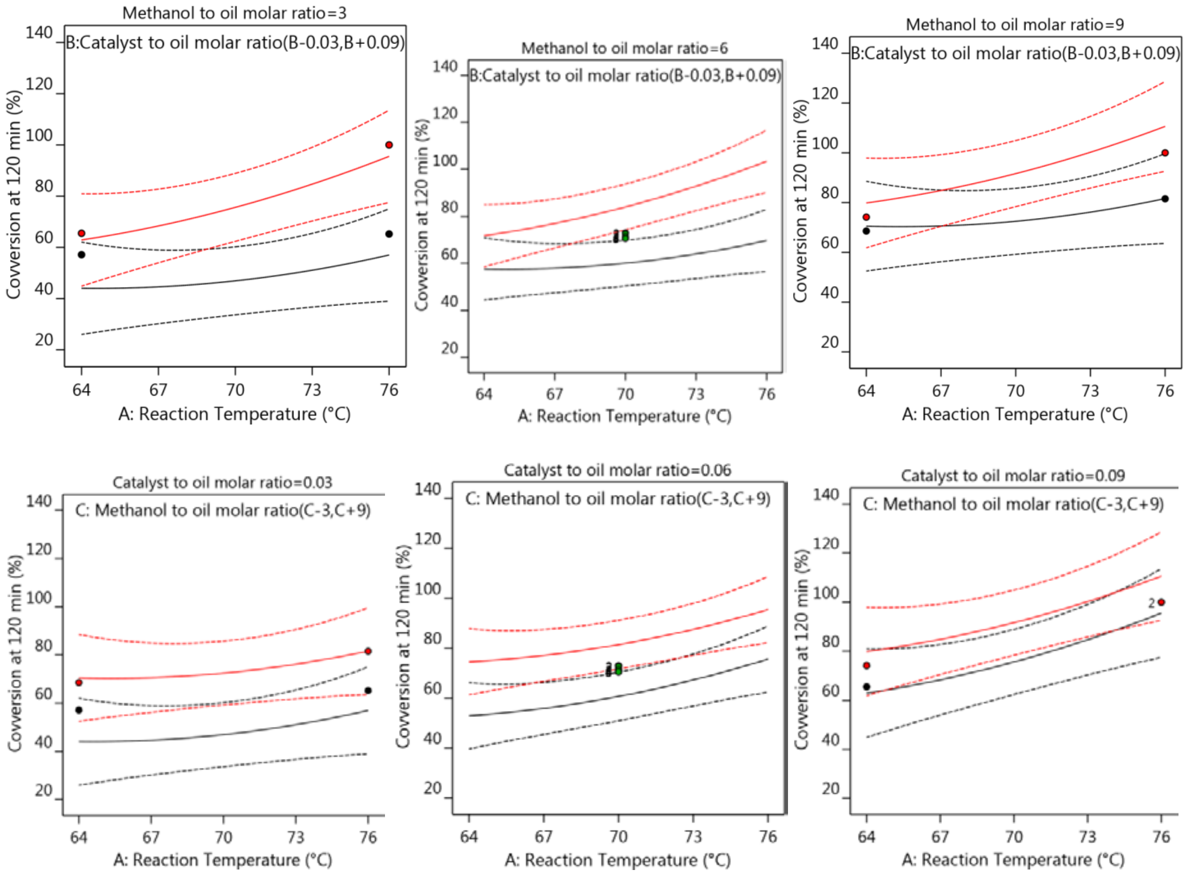

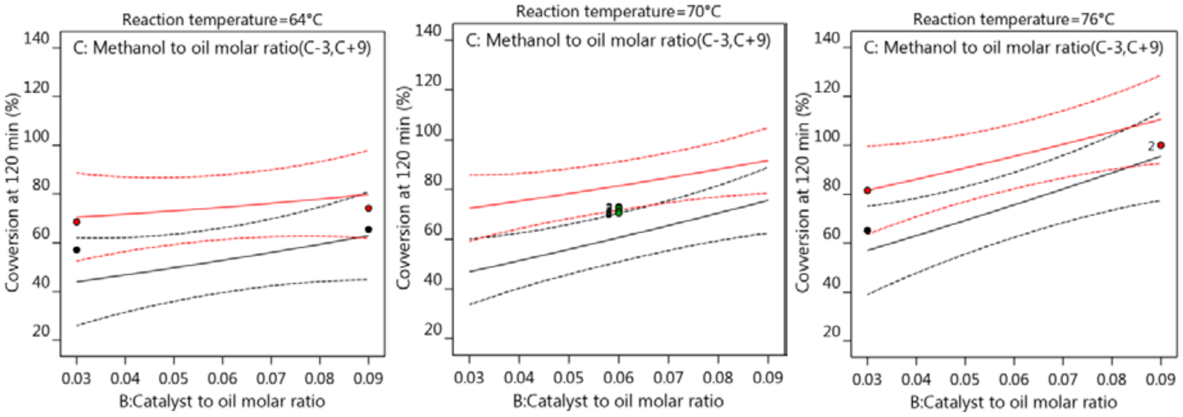

Figure S15. Interaction effects of factors on the conversion of triglyceride to FAME at 120 minutes of reaction time (a) Reaction temperature-Catalyst to oil molar ratio(B- 0.03 B+ 0.09),(b) Reaction temperature-Methanol to oil molar ratio(C- 3 ,C+ 9),(c) Catalyst to oil molar ratio- Methanol to oil molar ratio(C- 3,C+ 9).

S16 Analysis of the final biodiesel sample by ^{1}H NMR spectroscopy

The 1H NMR spectrum in Fig. S16(a) shows the chemical shifts of the signals of DBSA (Catalyst) and $CDCl_3$(Solvent)[1] where as Fig. S16(b) shows the chemical shifts of the signals of the various compounds of the reaction mixture. Fig. S16(c) depicted the spectrum of biodiesel at the end of the transesterification reaction (having achieved a conversion > 99%) after catalyst removal. The spectrum clearly indicates that no catalyst or glycerides remain in the final purified biodiesel and only FAME signals appears.

(a)

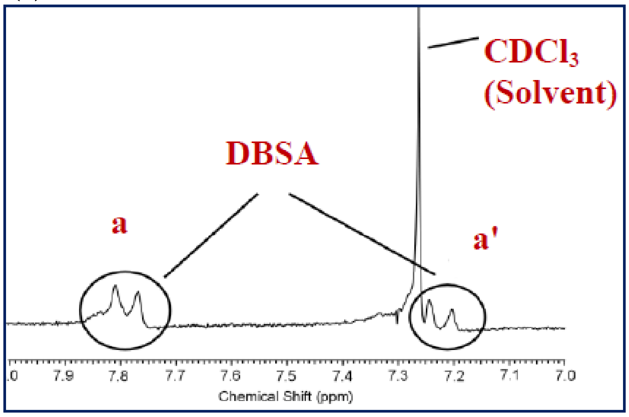

(b)

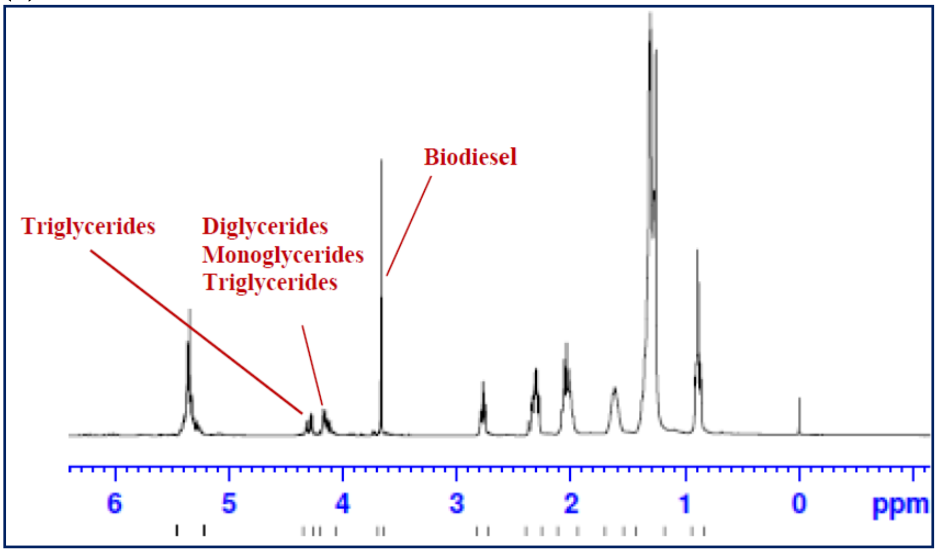

(c)

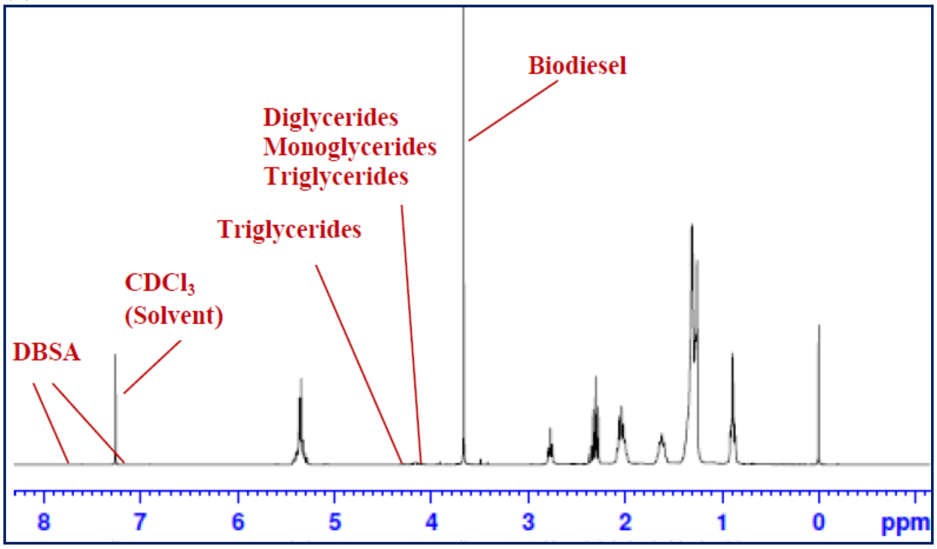

Figure S16. 1H NMR spectra (a) Catalyst(DBSA) (b) Biodiesel and glycerides (c) Purified biodiesel with no glycerides and catalyst..

- **7.0-8.0 ppm region (a and a') shows the chemical shifts in which signals due to the DBSA(catalyst)**
- **4.22-4.35 ppm region shows the chemical shifts in which signals due to the triglycerides**
- **4.07-4.22 ppm region shows the chemical shifts in which signals due to the triglycerides, diglycerides and mono-glycerides overlaps**
- **3.67 ppm region shows the chemical shifts in which signals due to the biodiesel**

References

1. Alegria, A., Fuentes, A. L., Arriba, D., Moran, J. R. & Cuellar, J. Biodiesel production using 4-dodecylbenzenesulfonic acid as catalyst. "*Applied Catal. B, Environ.* **160–161,** 743–756 (2014).
